# Toxicity evaluation of laser-synthesized pro-angiogenic carbon monoxide-rich gold nanoparticles *in vitro* and *in vivo*

**DOI:** 10.3389/fbioe.2025.1594693

**Published:** 2025-11-26

**Authors:** Thais Braga Gomes Araujo, Guilherme Conceição Concas, Mariana Gisbert, Gabriel de Farias Araujo, Leonardo da Cunha Boldrini Pereira, Lorena Gonçalves Henriques Corrêa Maduro, Lorena Oliveira Souza Soares, Sidney Fernandes Sales Junior, Davi Pinheiro Cunha, Fábio Veríssimo Correia, Anna Laurenzana, Cecilia Anceschi, Elena Frediani, Anastasia Chillà, Francesca Margheri, Tommaso Del Rosso, Enrico Mendes Saggioro

**Affiliations:** 1 Postgraduate Program in Public Health and the Environment, National School of Public Health, Rio de Janeiro, Rio de Janeiro, Brazil; 2 Laboratory for Environmental Health Assessment and Promotion, Oswaldo Cruz Institute, Rio de Janeiro, Rio de Janeiro, Brazil; 3 Laboratory of Laser Synthesis and Characterization of Nanomaterials, Department of Physics, Pontifical Catholic University of Rio de Janeiro, Rio de Janeiro, Rio de Janeiro, Brazil; 4 Division of Metrology in Biology (Dibio), Postgraduate Program in Translational Biomedicine, Postgraduate Program in Biotechnology, National Institute of Metrology, Quality and Technology (Inmetro), Xerém, Directorate of Applied Metrology for Life Sciences – Directorate of Scientific and Technological Metrology (Dimci), Duque de Caxias, Rio de Janeiro, Brazil; 5 Division of Metrology in Biology (Dibio), Postgraduate Program in Biotechnology, National Institute of Metrology, Quality and Technology (Inmetro), Xerém, Directorate of Scientific and Technological Metrology (Dimci), Duque de Caxias, Rio de Janeiro, Brazil; 6 Department of Natural Sciences, Federal University of the State of Rio de Janeiro, Rio de Janeiro, Rio de Janeiro, Brazil; 7 Department of Experimental and Clinical Biomedical Sciences, University of Florence, Florence, Italy

**Keywords:** nanotoxicology, zebrafish, ECFC, angiogenesis, carbon monoxide-rich gold nanoparticles

## Abstract

**Introduction:**

The safety profile of gold nanoparticles remains a concern and depends on dose, size, surface chemistry and charge. This study evaluated through *in vitro* and *in vivo* methods the cytotoxicity, oxidative stress and genotoxicity of laser synthesized carbon monoxide-Rich Gold Nanoparticles (COR-AuNPs), which can strongly promote angiogenesis in endothelial colony forming cells (ECFC).

**Methods:**

COR-AuNPs were synthesized by pulsed laser driven CO_2_ reduction reaction, and stabilized for culture media with the addition of the copolymer Pluronic-F127 (PF127). The fresh synthetized nanoparticles where characterized for size and morphology. Their stability was investigated in both culture media and the water aquarium environment. *In vitro* Cytotoxicity was assessed using the MTS assay on SaOS-2 cells and trypan blue staining in ECFCs at concentrations of 5 and 10 × 10^3^ μg.L^−1^. Zebrafish were exposed to a maximum concentration of 75 μg.L^−1^ of COR-AuNPs for 96 h. Oxidative stress biomarkers were assessed in liver and brain tissues, while genotoxicity was evaluated using the comet assay. The analyzed biomarkers included superoxide dismutase, catalase, reduced glutathione, total antioxidant capacity, carbonylated protein, and malondialdehyde.

**Results:**

The PF127 stabilized COR-AuNPs are stable in the zebrafish aquarium water for 6 weeks and showed no precipitation in the culture media. A strong pro-angiogenic activity was observed for ECFCs exposed to the COR-AuNPs at a minimal concentration of 5 × 10^3^ μg.L^−1^. The CO release is not immediate in the culture medium, suggesting that the COR-AuNPs are characterized by an intracellular release. No significant cytotoxicity was observed in both ECFC or SaOS-2 cells, and most oxidative stress biomarkers showed no significant effects in zebrafish. However, reduced glutathione levels decreased significantly in the brain at concentrations between 10 and 35 μg.L^−1^, likely due to the interaction with the metallic surface of the nanoparticles, while in the liver they increased significantly following exposure to COR-AuNPs at concentrations between 20 and 75 μg.L^−1^. No genotoxic effects were detected in zebrafish.

**Conclusion:**

COR-AuNPs enhance capillary morphogenesis in ECFCs, with minimal cytotoxicity, oxidative stress, and genotoxicity at sublethal concentration, supporting their safety for potential applications in regenerative therapies.

## 1 Introduction

The fast advancements in bio-nanotechnology have transformed various scientific fields, particularly biomedicine, by enabling the development of innovative materials with unprecedented therapeutic potential (Cabuzu et al., 2015). Among these, about two decades ago new kinds of metal-carbonyl structures denominated carbon monoxide release molecules (CORMs) were proposed to obtain the biological release of carbon monoxide (CO) in cells. The CORMS emerged as a promising treatment material due to their beneficial therapeutic properties (Motterlini and Otterbein, 2010). Although CO is widely known for its high toxicity (Omaye, 2002), due to its affinity for hemoglobin, which is approximately 200 times greater than that of oxygen (Ganong, 1995), it is also produced endogenously by enzymes such as heme oxygenase (Ryter et al., 2006), and plays a crucial role in biological processes, including anti-inflammatory (Tian et al., 2024), anti-apoptotic (Al-Huseini et al., 2014), anti-hypertensive (Penney and Howley, 1991) and antimicrobial activity (Cheng and Hu, 2021). Additionally, it can act as a protective agent in organ transplantation (Clark et al., 2003; Bagul et al., 2008; Nakao et al., 2008; Chen et al., 2009; Yoshida et al., 2010; Romão et al., 2012).

However, the therapeutic application of CO remains challenging, particularly when administered via inhalation (Choi et al., 2022; Yuan et al., 2022). These include a lack of tissue specificity and the need for specialized equipment to monitor *in vivo* CO levels (Queiroga et al., 2015), which can contribute to risks such as hypoxia and high toxicity responses (Omaye, 2002). To mitigate these risks, it is necessary to consider the application of CO within a narrow therapeutic window, typically limited to concentrations of up to 10% of COHb, as previously suggested in the literature (Omaye, 2002; Otterbein, 2009; Chu et al., 2021).

Most CORMs may act as carriers that facilitate the gradual and controlled release of CO. Beyond having a strong anti-inflammatory and antimicrobial effect (Mansour et al., 2022), CORMs have been deeply investigated in regenerative therapies, where the controlled release of CO acts as a signaling molecule that can regulate essential biological processes such as angiogenesis or osteodifferentiation in human stem cells (Volti et al., 2005; Fayad-Kobeissi et al., 2016; Diomede et al., 2020; Chen et al., 2021; Xu et al., 2023; Nguyen et al., 2024). The CORMs documented in the literature are based on transition metals (Cr, B, Fe, Mn, Co, Mo, Ru, W, Re, Ir) to which CO is covalently attached to form a metal-carbonyl complex (Herrmann, 1990; Motterlini et al., 2002; Nobre et al., 2007; Zhang et al., 2009; Crook et al., 2011; Kautz et al., 2016). This allows CO release through different mechanisms, including pH change, light irradiation, and ligand exchange (Schatzschneider, 2015). Despite the advantages offered by CORMs, their use as pharmaceutical agents presents a number of significant challenges (Otterbein, 2009). A notable disadvantage is that the composition of these compounds, which are based on transition metals such as manganese (Mn) and iridium (Ir), can result in adverse toxic effects within the body (Motterlini et al., 2002; Strigul et al., 2009). A previous study demonstrated that, even in the absence of direct cytotoxicity to cells, the metals used in CORMs can induce DNA damage, thereby posing a considerable risk to patients (Sawicka et al., 2022). Furthermore, many CORMs have to be stored at low temperatures, are chemically unstable in contact with air and have a low solubility in water, which creates several complications for their applicability and storage (Motterlini et al., 2002; 2005; Clark et al., 2003; Romão et al., 2012; Sawicka et al., 2022).

A promising approach to overcome these limitations has been proposed in a recent study, wherein has been demonstrated the feasibility of producing oxocarbon-rich gold nanoparticles by the pulsed laser driven CO_2_ reduction reaction (CO_2_RR), where the oxocarbons are constituted by carbon monoxide, formic, acetic, and lactic carboxylic acid (Tahir et al., 2024). A part of the produced nanomaterial is characterized by the presence of carbonyl groups covalently linked to the surface of the gold, being defined as carbon monoxide-rich gold nanoparticles (COR-AuNPs). COR-AuNPs has been recently proved promising for the release of CO *in vitro* e *in vivo* (Chillàet al., 2025), offering a potential alternative to the classical CORMs with highly toxic metal cores (Motterlini et al., 2002; Strigul et al., 2009). In fact, depending on the synthesis conditions, AuNPs are known for their stability in colloidal dispersion, resistance to temperature fluctuations, and low cytotoxicity, representing an ideal platform for a potential CO release (Balasubramanian et al., 2010; Howe et al., 2013). However, to date, no CORM with a gold metallic part has been synthesized by chemical protocols. Consequently, the COR-AuNPs represent a novel and interesting platform for CO delivery with potential clinical application. In this interesting perspective, as with any nanomaterial intended for a therapeutic use, their comprehensive toxicological assessment is essential to evaluate their biocompatibility and potential risks.

The zebrafish (*Danio rerio*) has emerged as an invaluable model organism in toxicological and biomedical research (Meyers, 2018). This species offers numerous advantages, including genetic and physiological similarities to humans (Balasubramanian et al., 2010), rapid embryonic development, high reproductive rates, and the transparency of its embryos (Kamstra et al., 2015). Additionally, zebrafish are easy to maintain and handle in laboratory environments, making them highly adaptable for a variety of experimental setups (Arunachalam et al., 2013). These unique characteristics make the zebrafish a versatile and reliable model for studying key toxicological endpoints such as oxidative stress and genotoxicity *in vivo* (Haque and Ward, 2018). Furthermore, their use has been particularly relevant in assessing the safety and biological effects of nanoparticles (Mutalik et al., 2024), reinforcing their applicability in nanotoxicology and biomedical research (Briggs, 2002; Kamstra et al., 2015).

One of the primary mechanisms underlying nanoparticle toxicity is oxidative stress, which results from an imbalance between the production of reactive oxygen (ROS) and nitrogen (RNS) species and the cellular antioxidant defense system (Mutalik et al., 2024). Excessive reactive species can trigger oxidative damage, leading to lipid peroxidation, protein carbonylation, and DNA strand breaks, all of which contribute to cytotoxic and genotoxic effects (Juan et al., 2021). Given the potential of nanoparticles to induce such responses, genotoxicity evaluations are crucial for understanding the broader implications of nanomaterial exposure (Shukla et al., 2021). Techniques such as the comet assay enable the detection of DNA damage at the cellular level, providing a comprehensive picture of the potential risks posed by nanoparticles (Mahaye et al., 2017). These combined assessments are essential for advancing nanomaterials toward safe biomedical applications. It is important to note that the intrinsic instability of CORMs presents a significant challenge in acquiring cytotoxicity data in complex organisms, creating a lack of information regarding interaction of living systems with these structures (Kautz et al., 2016). In this regard, biological models such as the zebrafish offer a promising approach for the evaluation of novel drugs and the assessment of their suitability for medical applications (Briggs, 2002; Kamstra et al., 2015; Meyers, 2018).

AuNPs, in particular, have been widely studied for biomedical applications due to their tunable physicochemical properties (Mallick et al., 2013) and biocompatibility (Connor et al., 2005). While some studies suggest that AuNPs are less toxic (Browning et al., 2009) and may exert neuroprotective effects (Torres et al., 2021), others have reported embryotoxic effects, including morphological abnormalities and developmental toxicity (Patibandla et al., 2018; Verma et al., 2018). Additionally, some works have highlighted the bioaccumulation of AuNPs in multiple tissues, including the brain, muscle, gills, and digestive tract, suggesting potential long-term toxicity (Geffroy et al., 2012). In this context, a comparative analysis of the literature, which will be presented in the following sections, can contribute to reveal that factors such as size, surface coating, and concentration play a crucial role in the biological response to AuNPs in studies *in vitro* and *in vivo* with the zebrafish model.

The present study aims to demonstrate different interesting aspects of the COR-AuNPs, such as their stability in complex biological media, their capacity to release CO, their safety profile and their strong pro-angiogenic effect in endothelial colony forming cells (ECFCs) at low minimal concentration, a function critical for tissue vascularization and that might find important applications also in the regeneration of bone tissues (Diomede et al., 2020; Xu et al., 2023). For this reason, we evaluated the degree of cellular toxicity in both ECFCs and bone-derived SaOS-2 cells, as well as oxidative stress and genotoxicity in zebrafish (*D. rerio*) exposed to sub-lethal concentrations. By combining these techniques, this research seeks to provide a critical understanding of the potential risks associated with the use of COR-AuNPs as a novel candidate for regenerative therapies.

## 2 Materials and methods

### 2.1 COR-AuNPs synthesis and characterization

The COR-AuNPs were obtained by pulsed laser driven CO_2_ reduction reaction with a gold target in water enriched with CO_2_ by NaOH addition, using an adapted method described elsewhere (Del Rosso et al., 2018; Tahir et al., 2024). In brief, a solution of NaOH at a concentration of 4 mmol.L^−1^ was prepared in deionized water. A gold target with a purity greater than 99%, supplied by Kurt J. Lesker, was positioned at the base of a Teflon beaker containing 8 mL of the prepared NaOH solution. PLA was performed for a duration of 6 hours in an open air-liquid interface, employing an Nd:YAG source (Q-Smart 850, Quantel United States) operating at a repetition rate of 10 Hz with a temporal width of 5.8 ns, wavelength of 532 nm, and at a fluence on the target of 3.5 J/cm^2^. Following the synthesis process, the amphiphilic copolymer Pluronic F-127 (PF127) was added to the colloidal dispersion reaching a final concentration of 2 mg. mL^−1^, to enhance the stability of the colloidal system in an environment with high ionic force or contaminants, such as ammonium compounds present in the system water used for acclimatization, and organic matter derived from fish food and biological debris released by the zebrafish themselves, as demonstrated in (Del Rosso et al., 2018).

The size distribution and morphology of the COR-AuNPs was evaluated by transmission electron microscopy (TEM) JEOL Model 2100F. An inductively coupled plasma mass spectrometer (Nexlon 300X Perkin Elmer, United States) was used to determine the metal concentration in the colloidal dispersion. The presence of the CO-Au bond was identified through the use of a micro-Raman spectrometer (HORIBA XploRA), with the sample previously dried on a glass slide to obtain the surface-enhanced Raman spectra (SERS), similarly as reported in (Muniz-Miranda et al., 2011). This technique is employed as a method to obtain the signal related to chemical bonds at remarkably low concentrations by exploiting the surface plasmon resonance of the COR-AuNPs, which leads to an enhancement of the electromagnetic field in a confined area near the COR-AuNPs surface. In optimal conditions, this approach can achieve an enhancement in sensitivity to single molecule levels (Kneipp et al., 1997), thus serving as a pivotal method for investigating low-concentration products.

The stability of COR-AuNPs in the zebrafish water environment was evaluated by monitoring the UV-Vis extinction spectra obtained by a double beam Lambda 950 Perkin Elmer spectrophotometer (United States). After the specimens were acclimatized, aquarium water was collected and used as the nanomaterial environment. The colloidal dispersion at concentration of 42 × 10^3^ μg.L^−1^ was added to the fresh water (50:50 v/v), and the UV-Vis spectra was monitored for 6 weeks at room temperature. The colloidal stability of the COR-AuNPs in cell culture medium was evaluated for 24 h by Dynamic Light Scattering measurement using the intensity distribution as a function of the hydrodynamic diameter. The analysis was carried out at 25 °C using an SZ-100 analyzer from Horiba Instruments (Kyoto, Japan), with a scattering angle of 90°, using a 10 mW laser with a wavelength of 532 nm. For the analysis, the COR-AuNPs were dispersed in a phosphate buffer solution (PBS) with pH 7.4, and RPMI + 10% FBS culture medium at ratio of 3:1 (nanoparticle:culture medium/PBS). COR-AuNPs containing 2 mg.mL^−1^ of the amphiphilic block copolymer Pluronic-F127 (PF127) were used for the test.

### 2.2 COR-AuNPs cytotoxicity in SaOS-2 and ECFCs

The SaOS-2 cell line (BCRJ-0217) was obtained from the Rio de Janeiro Cell Bank (https://bcrj.org.br/) packed in frozen ampoules and kept in liquid nitrogen. After thawing, cells were grown in McCoy’s 5A medium (Sigma-Aldrich) supplemented with 10% FBS (BioNutrientes) in an incubator at 37 °C in a humidified environment (CellXpert^®^ C170i, Eppendorf, Hamburg, Germany) with 5% CO2 atmosphere. Sterility was ensured by tests for bacteria, fungi, and mycoplasmas. For bacteria and fungi, the cell culture supernatant was placed in thioglycolate (TIO) (Acumedia, Baltimore, United States) and tryptic soy broth (TSB) (Acumedia, Baltimore, United States) and incubated aerobically for 14 days at 22.5 °C ± 2.5 °C and 32.5 °C ± 2.5 °C, respectively. *Mycoplasma* contamination in the cell supernatants was investigated by bioluminescence using the MycoAlertTM PLUS *Mycoplasma* Detection Kit (MycoAlert^®^, Lonza, Verviers, Belgium) and all samples were confirmed to be free of contamination. The cytotoxicity was evaluated according to ISO 19007:2018 – *in vitro* MTS assay for measuring the cytotoxic effect of nanoparticles. SaOS-2 cells were seeded in 96-well plates at a density of 3 × 10^4^ cells per well corresponding to a density of about 93.750 cells per cm^2^ (each well ∼ 0,32 cm^2^) and maintained for 24 h in McCoy’s 5A medium (Sigma-Aldrich) supplemented with 10% FBS (BioNutrientes) in an incubator at 37 °C with a 5% CO_2_ atmosphere. Subsequently, the cells were treated with COR-AuNPs at concentrations of 5 and 10 × 10^3^ μg.L^−1^. After 24 h of exposure, the culture medium was discarded, and 200 µL of 0.01 M PBS (Sigma-Aldrich) was added (3×) to wash the cells and remove excess nanoparticles. To assess cell viability, the commercial CellTiter 96^®^ AQueous Non-Radioactive Cell Proliferation Assay (MTS) (Promega) was used. The MTS reagent was prepared in phenol red-free culture medium, and 120 µL was added to each well across the plate. The incubation time was 1 h at 37 °C, after which absorbance was measured at 490 nm using a Biotek Synergy H4 microplate reader (Aligent, Santa Clara, California, United States). After the initial absorbance reading, the supernatant was collected, subjected to centrifugation at 25.155 RCF for 90 min, and absorbance was measured again. This step was designed to minimize potential interferences. There was no significant difference between the readings obtained before and after centrifugation. The negative control consisted of cells cultured in complete medium alone (vehicle). The positive control was performed with 20% DMSO to induce cell death.

Endothelial Colony Forming Cells (ECFCs) were isolated from Human Umbilical Cells (>50 mL) of healthy newborns, as described elsewhere (Margheri et al., 2011; Armanetti et al., 2021), with informed consent from the mothers (R711-D) and observing the Italian legislation (art. 2, paragraph 1, letter f, decree of 18 November 2009). The cells were analyzed by flow-cytometry for expression of the antigens CD45, CD34, CD31, CD105, ULEX, vWF, KDR, and uPAR. ECFCs were cultured in 10% fetal bovine serum (FBS, Euroclone) supplemented EGM2 cell culture media, incubated at 37 °C with 5% CO_2_ saturation.

ECFCs were seeded in 6 cm dishes at a density of 2 × 10^4^ cells/cm^2^ and cultured in a humidified incubator at 37 °C with 5% CO_2_. After cell attachment, they were incubated with EGM-2 medium (3 mL/well) containing COR-AuNPs at concentrations of 5 × 10^3^ and 10 × 10^3^ μg·L^−1^ for 24 h. Cytotoxicity was determined by trypan blue staining: 20 µL of cell suspensions were resuspended with an equal volume of 0.4% (w/v) trypan blue solution prepared in 0.81% NaCl and 0.06% (w/v) dibasic potassium phosphate. Viable and non-viable cells (trypan blue positive) were counted separately using a dual-chamber hemocytometer and a light microscope. Optical microscopy was used to assess qualitative intracellular uptake of COR-AuNPs. Due to their strong light-scattering properties and high electron density, AuNPs appear as dark, punctate regions within the cytoplasm under brightfield illumination. Cellular images were acquired through the EVOS xl core microscope (AMG, Advanced Microscopy Group).

### 2.3 Intracellular CO release measurement

To assess carbon monoxide (CO) release from the nanoparticles, we quantified carboxyhemoglobin (COHb) levels using a specific ELISA assay. COHb, formed by the binding of CO to hemoglobin, serves as a reliable indicator of intracellular CO presence. For this purpose, we employed human chronic myeloid leukemia K562 cells (DSMZ), which were induced toward erythroid differentiation using 1 µmol.L^−1^ Imatinib for 5 days (Jacquel et al., 2003). These cells act as erythroid progenitors capable of synthesizing hemoglobin, providing a biologically relevant model for evaluating CO delivery. Following the treatment, the cells were exposed to 15 × 10^3^ μg.L^−1^ of COR-AuNPs, or to 100 μM solution of CORM-2 (Sigma-Aldrich). Cell lysates were analyzed using the COHb ELISA kit (96-well format, MyBioSource, MBS7254040) according to the manufacturer’s instructions, enabling sensitive and specific detection of CO-bound hemoglobin within a cellular context that mimics erythropoiesis. The instructions include a final centrifugation process (at 1000 RCF for 15 min at 2 °C–8 °C), which eliminate the potential interference of the extinction of the AuNPs in the measurement.

### 2.4 COR-AuNPs *in vitro* capillary morphogenesis

*In vitro* capillary morphogenesis was performed in tissue culture wells with Matrigel (BD Biosciences) coating. A total of 18.000 cells were seeded per well, corresponding to a density of 60.000 cells/cm^2^ (well growth area: 0.3 cm^2^) in 2% FBS supplemented EGM-2 culture medium, and incubated at 37 °C at 5% CO2 atmosphere. The pictures were acquired at regular intervals at EVOS optical microscope (Thermo Fisher Scientific, Monza, Italy). The Angiogenesis Analyzer tool of ImageJ software19 provided the statistical analysis for each experimental condition tested quantifying nodes, master junctions and meshes Nodes are identified as pixels that have at least three neighbors, corresponding to a bifurcation. Master junctions are element junctions linking at least three segments. They delimited the master segments”. Meshes are the polygon structures reinforced with more than one layer of cells in their walls and have also been referred to by other authors as a “Honeycomb formation”.

### 2.5 Set-up for zebrafish acclimatization and COR-AuNPs exposure

This study was approved by the Animal Use Ethics Committee of the Oswaldo Cruz Institute (protocol 035/2022 and license L-001/2023-A1, valid until 2026). Adult zebrafish (*D. rerio*) used in the experiments had an average weight of 0.4 g and length of 3.5 cm. Fish were maintained in glass aquaria with dechlorinated water (pH 7.0–7.6; 23 °C–27 °C), under controlled ammonia levels and a 14:10 h light:dark photoperiod. They were fed with Alcon^®^ Neon feed (Alcon, Camboriú, Brazil) via automatic digital feeders. Acclimation lasted 7 days, in accordance with ABNT NBR 15088 guidelines (ABNT NBR, 2022).

Following acclimation, males and females (4.17 ± 0.35 cm; 2.26 ± 0.45 g) were randomly transferred to 36 L exposure aquaria (49.5 × 30 × 24.5 cm), at a density of 1 g of fish per liter. Eighteen fish were placed in each tank, with three replicates per group, totaling 324 animals. Six fish per group were allocated for genotoxicity analysis via the comet assay.

For the acute exposure test, a COR-AuNPs stock dispersion was serially diluted to obtain final concentrations of 5, 10, 20, 35, and 75 µg·L^−1^. All concentrations were sublethal and based on previous toxicological data. Souza et al. (2021) reported complete lethality in zebrafish at concentrations >300 µg·L^−1^ of gold nanorods (AuNRs), while 75 µg·L^−1^ was considered sublethal. Thus, 75 µg·L^−1^ was adopted as the highest test concentration. Additionally, Gong et al. (2016) demonstrated physiological effects without lethality at 60 µg·L^−1^ of cobalt in CO-releasing molecules (CORMs), supporting the sublethal design of this experiment. Despite differences in metal core composition, the common mechanism of CO release ensures biological comparability and relevance.

The exposure period lasted 96 h, with solution renewal at 48 h to maintain contaminant levels, establishing a semi-static system (Ge et al., 2015). Control groups were maintained under identical conditions, but without COR-AuNPs.

At the end of the 96-h exposure, fish were euthanized in accordance with ethical guidelines established by the Brazilian National Council for the Control of Animal Experimentation (CONCEA, 2015), and aligned with international standards (Wendy Underwood, 2020; National Cancer Institute, 2023). The procedure was performed by immersion in an ice-water solution (5:1 ratio), maintained between 0 °C and 4 °C. Death was confirmed by the absence of opercular (gill) movements, and the animals were kept in the solution for at least 10 min to ensure complete loss of vital signs. This method was chosen to avoid the use of chemical anesthetics, which could interfere with subsequent biochemical analyses, as previously described by Borski and Hodson (2003).

### 2.6 Tissue collection and homogenization

After euthanasia, liver and brain samples were collected, and 30 mg of each tissue were selected. A total of 1,500 µL of 50 mmol·L^−1^ potassium phosphate buffer (pH 7.0) was added. The buffer was prepared using ultrapure water, monobasic potassium phosphate (KH_2_PO_4_; Sigma-Aldrich, St. Louis, MO, United States) and dibasic sodium phosphate (Na_2_HPO_4_; Merck, Darmstadt, Germany). The tissue was homogenized for 60 s using a stainless steel spatula. Samples were then centrifuged at 9.400 RCF for 10 min at 4 °C using a 5430R centrifuge (Eppendorf, Hamburg, Germany). The resulting supernatant was transferred to new microtubes (Eppendorf, Hamburg, Germany) and stored at −80 °C until further analysis. The protocol was adapted from Yan et al. (2015) and Yan et al. (2016).

### 2.7 Antioxidant and oxidative stress biomarkers

#### 2.7.1 Total protein evaluation

Total protein quantification was performed to standardize the analysis of enzymatic and non-enzymatic biomarkers. The procedure was adapted from Lowry et al. (1951), with modifications by Peterson et al. (2008). After extraction, 20 µL of the resulting sample was mixed with 980 µL of ultrapure water, followed by the addition of 400 µL of reagent A, prepared using sodium hydroxide (NaOH; Sigma-Aldrich, St. Louis, MO, United States), sodium dodecyl sulfate (SDS; Merck, Darmstadt, Germany), and CTC solution containing sodium carbonate (Sigma-Aldrich), sodium potassium tartrate (Isofar, Duque de Caxias, Brazil), and copper sulfate (Dinâmica, Indaiatuba, Brazil), all diluted in ultrapure water. Next, 200 µL of 2N Folin–Ciocalteu reagent (Sigma-Aldrich) was added, and the mixture was incubated in the dark for 30 min. Absorbance was measured at 750 nm using an LTEK INNO microplate reader (Gyeonggi-do, South Korea), and results were calculated based on a BSA calibration curve (bovine serum albumin; Sigma-Aldrich). Data are expressed in µg·µL^−1^.

#### 2.7.2 Superoxide dismutase evaluation (SOD)

The SOD biomarker was quantified using the Superoxide Dismutase Assay Kit (Cayman Chemical Company, Ann Arbor, MI, United States). The reaction mixture consisted of 200 µL of radical detector, 50 µL of liver or brain extract, and 20 µL of xanthine oxidase. The calibration curve was prepared with SOD standards, and absorbance readings were performed at 450 nm using an LTEK INNO microplate reader (Gyeonggi-do, South Korea). Results are expressed as international units of enzymatic activity (U) normalized to protein content (U·g protein^−1^).

#### 2.7.3 Catalase evaluation (CAT)

Catalase (CAT) activity was determined following the protocol by Aebi (1984). Liver samples were diluted 1:10 by mixing 410 µL of sample with 3,690 µL of ultrapure water; brain samples were not analyzed due to low enzyme activity (Gomes et al., 2019; Araujo et al., 2022). Hydrogen peroxide solution was prepared using hydrogen peroxide (Merck, Darmstadt, Germany) and 50 mmol·L^−1^ potassium phosphate buffer (pH 7.0). Enzyme kinetics were measured by monitoring the decrease in absorbance of H_2_O_2_ at 240 nm (ΔAbs min^−1^) over 15 s using a UV-Vis spectrophotometer (MS1 model, Bel Photonics, São Paulo, Brazil). Quartz cuvettes were used as blanks and samples. Results are expressed as international units of enzymatic activity (U) per gram of protein.

#### 2.7.4 Reduced glutathione evaluation (GSH)

The GSH concentration was determined using the method established by Wilhelm Filho et al. (2005), with adaptations made for use with a microplate reader. The calibration curve was prepared by adapted for use with a microplate reader. The calibration curve was prepared with concentrations ranging from 0 to 300 µmol·L^−1^ from a stock solution of L-Glutathione Reduced (Sigma-Aldrich, St. Louis, MO, United States). A 2x dilution was performed by mixing 400 µL of sample with 400 µL of ultrapure water. For analysis, 350 µL of each calibration standard or sample was mixed with 350 µL of 5,5′-dithiobis (2-nitrobenzoic acid) (DTNB; Sigma-Aldrich), incubated in the dark for 15 min, and absorbance was measured at 412 nm using an LTEK INNO microplate reader (Gyeonggi-do, South Korea). Results are expressed in nmol per milligram of protein.

#### 2.7.5 Glutathione-S-Transferase evaluation (GST)

Glutathione S-transferase (GST) activity was determined by measuring the formation of 2,4-dinitrophenyl glutathione (GS-DNB), the product of the conjugation of L-Glutathione Reduced (Sigma-Aldrich, St. Louis, MO, United States) with 1-chloro-2,4-dinitrobenzene (CDNB; Sigma-Aldrich). The method was adapted from Habig et al. (1974). Absorbance was monitored at 340 nm using a UV-Vis spectrophotometer (MS1 model, Bel Photonics, São Paulo, Brazil) over 60 s. Reactions were performed in quintuplicate with different dilutions for liver and brain samples. The reaction mixture was prepared by combining sample aliquots with ultrapure water, phosphate buffer, L-Glutathione Reduced, and CDNB to prevent premature reaction prior to measurement. Enzymatic activity was calculated based on the formation of 1 µmol of GS-DNB per minute and expressed as GST units per gram of protein.

#### 2.7.6 Total antioxidant capacity evaluation (TAC)

Total antioxidant capacity (TAC) was measured using the Trolox equivalent antioxidant capacity (TEAC) method. Phosphate-buffered saline (PBS; 5 mmol·L^−1^, pH 7.4) was prepared using potassium dihydrogen phosphate (KH_2_PO_4_; Sigma-Aldrich, St. Louis, MO, United States) and dipotassium hydrogen phosphate (K_2_HPO_4_; Merck, Darmstadt, Germany). Solutions of ABTS (7 mmol·L^−1^; Merck) and potassium persulfate (2.45 mmol·L^−1^; Sigma-Aldrich) were prepared, and mixed the day before the analysis to form a concentrated solution stored in the dark. On the day of analysis, the mixture was diluted with PBS to an absorbance of 0.700 ± 0.020 at 734 nm and 30 °C, adjusting as necessary. Trolox standard curves (0–0.75 mmol·L^−1^) were constructed. Samples (10 µL of diluted supernatant) were added to the ABTS/potassium persulfate mixture in a microplate, and absorbance was measured at 734 nm and 30 °C using an LTEK INNO microplate reader (Gyeonggi-do, South Korea). Results were calculated by linear regression of the standard curve.

#### 2.7.7 Carbonylated protein evaluation (CPT)

Carbonylated protein (CPT) levels were quantified following the protocol of Mesquita et al. (2014). Briefly, 84 µL of supernatant from each organ (liver and brain) was diluted 1:5 with ultrapure water. In the microplate, 80 µL of 2,4-dinitrophenylhydrazine (DNPH; 10 mmol·L^−1^; Dinâmica, Indaiatuba, Brazil) and 80 µL of sample were added, followed by incubation at room temperature for 10 min. Then, 40 µL of 6 mol·L^−1^ sodium hydroxide (NaOH; Sigma-Aldrich, St. Louis, MO, United States) was added, the mixture homogenized by pipetting, incubated for another 10 min, and absorbance read at 450 nm using an LTEK INNO microplate reader (Gyeonggi-do, South Korea). Blanks consisted of 80 µL sample plus 80 µL of 0.5 mol·L^−1^ phosphoric acid (H_3_PO_4_; Vetec, Duque de Caxias, Brazil), processed identically. Results are expressed as µmol carbonylated protein per gram of tissue, normalized per gram of protein (µmol·g^−1^ tissue per g protein).

#### 2.7.8 Malondialdehyde evaluation (MDA)

Malondialdehyde (MDA) levels were quantified using the TBARS assay kit (Cayman Chemical Company, Ann Arbor, MI, United States) following the manufacturer’s protocol. For quantification, 80 µL of supernatant from each organ (liver and brain) was mixed with 80 µL of ultrapure water, followed by the addition of 150 µL of 10% trichloroacetic acid (TCA; Merck, Darmstadt, Germany) and 1,200 µL of 0.53% thiobarbituric acid (TBA; Merck). The TBA solution was prepared by diluting TBA in a 1:1 solution of 20% acetic acid (Merck) and 0.7 mol·L^−1^ sodium hydroxide (NaOH; Merck). Calibration curves were constructed using MDA standards (1–70 µmol·L^−1^) prepared in ultrapure water and treated identically. Samples and standards were incubated at 90 °C for 1 h in a thermoblock (Benfer B-BTB, São Paulo, Brazil), followed by an ice bath for 10 min and centrifugation at 1,600 × g for 10 min at 4 °C using an Eppendorf 5430R centrifuge (Hamburg, Germany). Absorbance was measured at 535 nm using an LTEK INNO microplate reader (Gyeonggi-do, South Korea). Results are expressed as µmol·L^−1^ MDA normalized per gram of protein (µmol·L^−1^ per g protein).

### 2.8 Comet assay

Genotoxicity was assessed using the alkaline comet assay, adapted from OECD Test Guideline 489 for the zebrafish (*D. rerio*) model (OECD Science, 2016). Six fish per experimental group were used, and blood was collected by caudal vein puncture, forming pools by contaminant concentration. Each pool (10 µL) was mixed with 3 µL of heparin (Parinex^®^; Hipolabor, Belo Horizonte, Brazil) and embedded in low melting point agarose (LMP agarose; Sigma-Aldrich, St. Louis, MO, United States) at 37 °C. The mixture was applied onto slides previously coated with normal melting point agarose (NMP agarose; Sigma-Aldrich) at 60 °C. After solidification (4 °C ± 2 °C), slides were lysed for 24 h at 4 °C ± 2 °C in a buffer containing sodium chloride (2.5 mol·L^−1^; Synth, Diadema, Brazil), EDTA (0.1 mol·L^−1^; Vetec, Duque de Caxias, Brazil), Tris (0.01 mol·L^−1^; Sigma-Aldrich), Triton X-100 (Sigma-Aldrich), and dimethyl sulfoxide (DMSO; Dinâmica, Indaiatuba, Brazil). Electrophoresis was conducted in alkaline buffer (10 mol·L^−1^ sodium hydroxide and 0.2 mmol·L^-1^ EDTA) at 0 °C–4 °C, with 20 min of DNA unwinding followed by 20 min of electrophoresis at 25 V and 300 mA. Slides were then neutralized with PBS (pH 7.4), fixed in absolute ethanol (Dinâmica), stained with silver nitrate (Sigma-Aldrich), and dried at room temperature. Comet images were captured using an optical microscope (Kasvi–Motic, ×400 magnification) and analyzed using Motic Image Plus 2.0^®^ software. At least 200 nucleoids per slide (five slides per group) were evaluated. DNA damage was assessed based on tail intensity, tail moment, and classified using a five-grade scale (0–4), according to Noroozi et al. (1998). Negative controls consisted of animals maintained in aquarium water under the same experimental conditions, without exposure to COR-AuNPs (see Supplementary Figure S1 in Supplementary Material). Although a positive control was not included in this experiment, our group routinely validates the assay using methyl methanesulfonate (MMS; Sigma-Aldrich) in various models, ensuring methodological reliability. This assay was performed following a protocol previously validated by our group (Sales Junior et al., 2020; 2021; 2024), in accordance with the Minimum Information for Reporting on the Comet Assay (MIRCA) guidelines (Møller et al., 2020), ensuring reproducibility and transparency of the results (Supplementary Table S1 on Supplementary Material).

### 2.9 Statistical analysis

All statistical analyses were performed using GraphPad Prism software (version 9.5.1; GraphPad Software, San Diego, CA, United States). Data normality was assessed using the Shapiro–Wilk test (α = 0.05). For datasets with normal distribution, one-way ANOVA followed by Dunnett’s multiple comparisons test was applied. In cases where normality was not confirmed, non-parametric analyses were conducted using the Kruskal–Wallis test followed by Dunn’s *post hoc* test. All biomarker datasets from the zebrafish experiments failed to meet the normality assumption and were therefore analyzed using non-parametric methods. Results are expressed as median ± standard error of the mean (SEM), and statistical significance was set at p < 0.05. For the SaOS-2 assays, data normality was verified using the Shapiro–Wilk test, followed by one-way ANOVA with multiple comparisons. Results are presented as mean percentage values relative to non-exposed control wells (considered as 100%), which were cultured in medium alone.

## 3 Results and discussion

### 3.1 COR-AuNPs characterization and CO release

**FIGURE 1:**
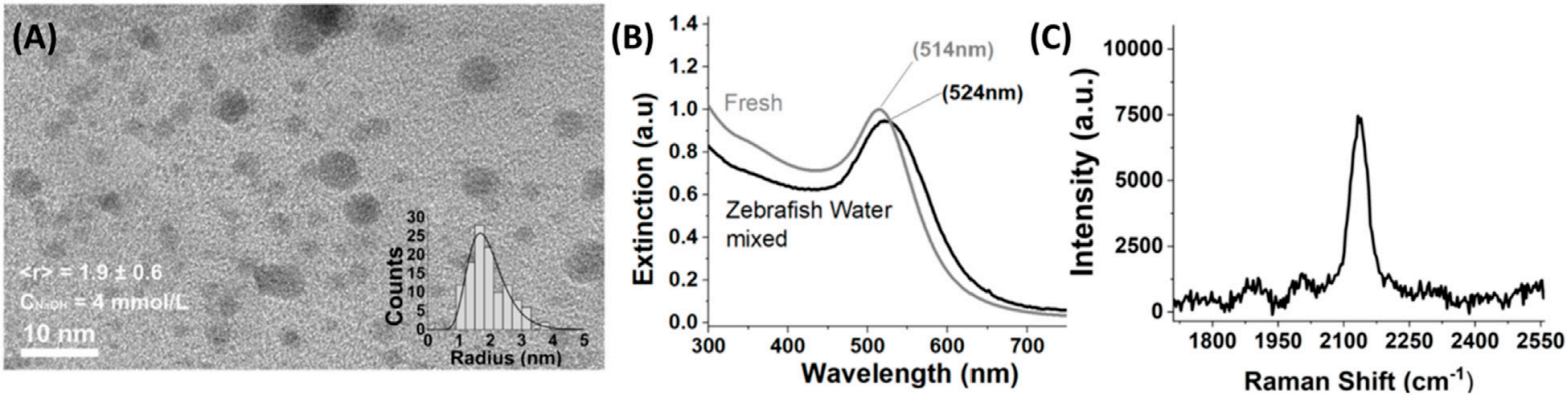
**(A)** TEM images of the colloidal dispersions of COR-AuNPs synthesized by PLA at air-water interface with a concentration of NaOH (C_NaOH_) of 4 mmol.L^−1^. In the inset is represented the statistical size distribution. **(B)** UV-Vis spectra of the colloidal dispersions of COR-AuNPs before (fresh-grey line) and after the addition of PF127 followed by the incubation in the zebrafish aquarium. **(C)** SERS spectra obtained from the analysis of a few drops of COR-AuNPs dried on silicon substrate. The Au-CO peak (2,124 cm^−1^) is highlighted.

The PLA process resulted in the formation of well-defined spherical nanoparticles with a narrow distribution, measuring less than 10 nm (Figure 1A). As the synthesis process was performed in batch without a water flowing system, the nanoparticles were also subjected to laser fragmentation in liquid subsequent to their production, resulting in the formation of ultrasmall nanoparticles with an average radius of 1.9 nm, as previously reported (Tahir et al., 2024).

As stated in the introduction, the pulsed laser driven CO_2_ reduction reaction results to the synthesis of gold nanoparticles rich in CO (Del Rosso et al., 2016) and organometallic nanocomposites constituted by gold nanoclusters embedded in formic, acetic and lactic acids, with concentrations of about 0.3, 1.4, and 0.2 ppm, respectively (Tahir et al., 2024). The UV-Vis spectra of the pristine colloidal dispersion of nanoparticles exhibit the characteristic localized surface plasmon resonance (LSPR) peak at 514 nm, which shifts to 524 nm after the addition of the amphiphilic copolymer block Pluronic-F127 (PF127) and the subsequent incubation in the zebrafish aquarium. As visible in Figure 1B, the shape and full width half maximum (FWHM) of the LSPR curve is not affected by the zebrafish water environment, and we only notice a slight decrease in the intensity of the maximum, probably associated to a small portion of nanoparticles (less than 10%) precipitated after the incubation in the aquarium. The SERS measurement demonstrated the existence of gold-carbonyl (Au-CO) chemical bonds, thereby confirming the presence of CO associated with the COR-AuNPs (Figure 1C).

**FIGURE 2:**
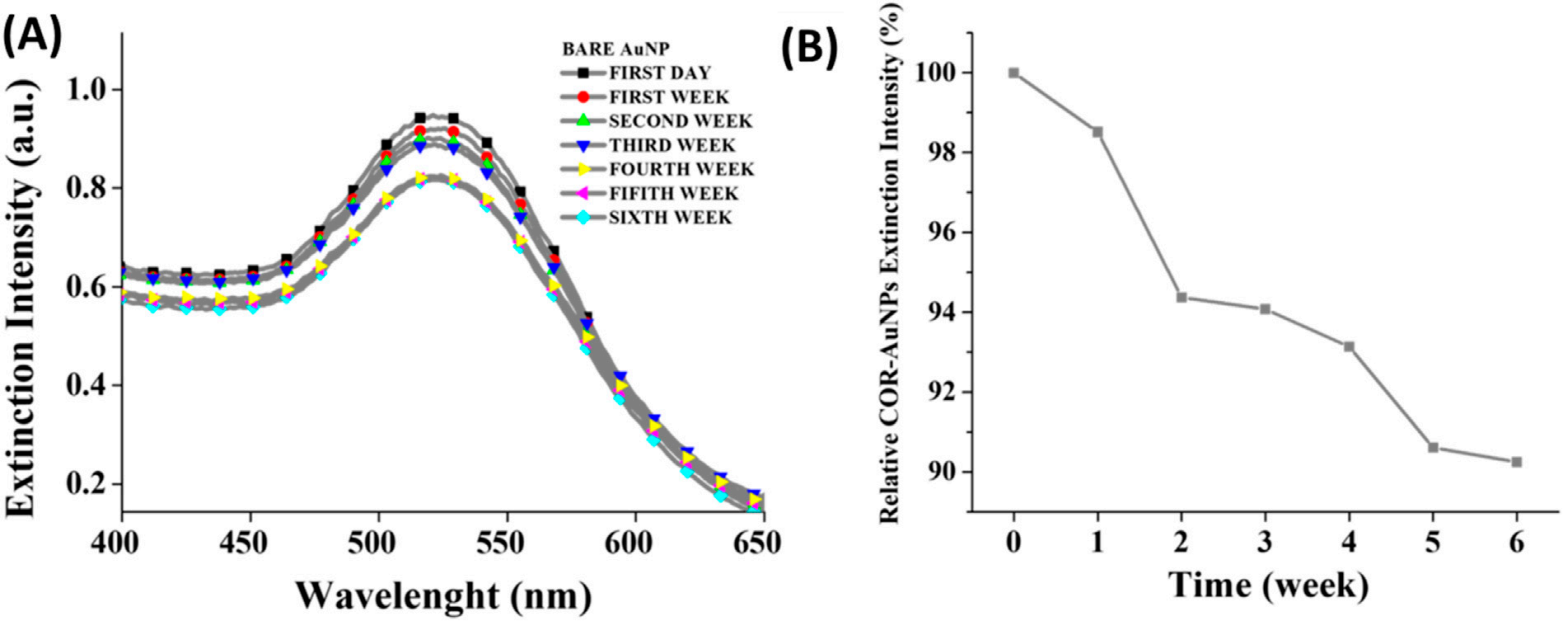
Monitoring of the extinction spectra **(A)** and of the relative peak intensity at the wavelength of 525 nm **(B)** of the COR-AuNPS with PF127, after exposure to the natural zebrafish water over a period of 6 weeks.

The copolymer PF127 was utilized as a method to enhance the stability of the COR-AuNPs, as demonstrated in previous study (Del Rosso et al., 2018). In addition to the afforementioned effect, PF127 has applications as a drug delivery system (Moore et al., 2000), facilitating the penetration of nanocomposites into cancer cells (Elagin et al., 2014; Simon et al., 2015). In this study, we assess the stability of the NPs in zebrafish aquarium water by monitoring the variation in intensity of the extinction peak of the COR-AuNPs (Figure 2A) over a 6-weeks period. It can be observed that the NPs were markedly stable, with the peak extinction reducing by only 6% over the course of the experiment (1 week), and by only 10% within 6-weeks (Figure 2B), while the FWHM had a variation of 0.8% and 7.6% indicating minimal agglomeration after their dispersion in the zebrafish water (Sangwan and Seth, 2022). The physicochemical stability of the COR-AuNPs is a fundamental requirement for their applicability in biological systems (Sharma et al., 2021), as instability can lead to aggregation, which in turn alters the nanoparticle’s biodistribution, bioavailability, and potential toxicity (Abbasi et al., 2023).

A dynamic light scattering (DLS) analysis of COR-AuNPs in phosphate buffered saline (PBS) and McCoy’s 5A cell culture medium supplemented with fetal bovine serum (FBS) measurements were taken at 0 h for both conditions, and after 24 h for COR-AuNPs in McCoy’s 5A + FBS. Peaks centered at 13 nm and 49 nm were observed for COR-AuNPs in PBS and cell culture medium, respectively, indicating a possible interaction with FBS. Following a 24-hour period of incubation in cell medium, we observed a small shift of the peak to 51 nm, indicating that the COR-AuNPs functionalized by PF127 are stable in complex biological media (Supplementary Figure S2 on Supplementary Material).

**FIGURE 3:**
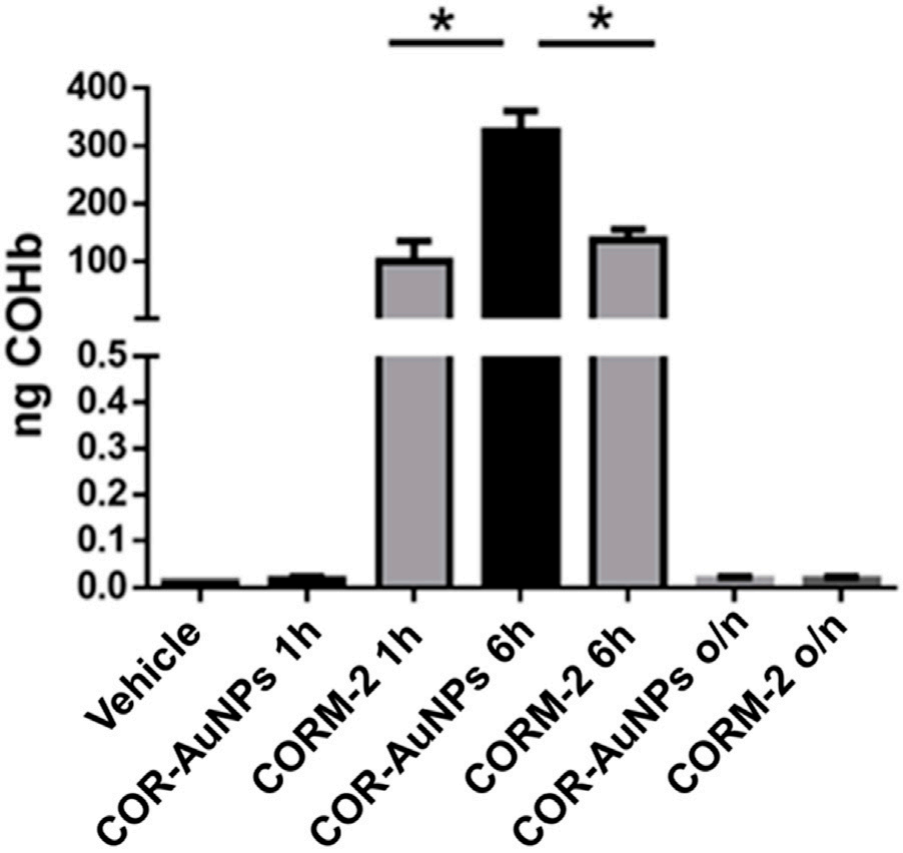
Quantification of COHb released by CORM-2 (100 μM) and COR-AuNPs (20 × 10^3^ μg.L^−1^) in human chronic myeloid leukemia cells. o/n: over night.

The characterization of the COR-AuNPs was completed by measuring the release of CO in cells using the Elisa COHb test on human chronic myeloid leukemia cells (K562). The results of CO release are reported in Figure 3, showing the formation of COHb when K562 cells were treated with both CORM-2 and COR-AuNPs. The CO release profiles exhibited significant variation over the duration of one hour. The release rate of CORM-2 was found to be accelerated, and after a duration of 6 h, the concentration of COHb was observed to be elevated in the presence of COR-AuNPs. The ensuing results illustrate the disparities in the time-dependent mechanisms of CO release for the structures in question. Indeed, extant literature on the subject indicates that the release of carbon monoxide (CO) by CORM-2 occurs extracellularly and commences within minutes of its dispersion in the culture medium (Southam et al., 2021). Conversely, the COR-AuNPs necessitated a duration exceeding 60 min for the identification of COHb, thereby indicating an intracellular initiation of the CO release process.

The laser-synthesized COR-AuNPs presented herein offer several advantages over most commercial CORMs that frequently contain toxic metals (e.g., Cr, B, Fe, Mn, Co, Mo, Ru, W, Re, Ir) (Herrmann, 1990; Motterlini et al., 2002; Nobre et al., 2007; Zhang et al., 2009; Crook et al., 2011; Kautz et al., 2016), require protection from light, must be stored at −20 °C (Pizarro et al., 2009; Crook et al., 2011), and present low solubility in water (Motterlini et al., 2002; Bannenberg and Vieira, 2009). In contrast, the synthesis of COR-AuNPs employs a one-step, eco-friendly technique that utilizes solely gold, water and aqueous CO_2_. These nanoparticles exhibit stability at room atmosphere and can be stored at room temperature on the shelf, as evidenced by the COHb levels measured after a few months of storage, that remained consistent, indicating the preservation of the CO release property (Chillàet al., 2025).

### 3.2 Cytotoxicity and pro-angiogenic activity of the COR-AuNPs *in vitro*

**FIGURE 4:**
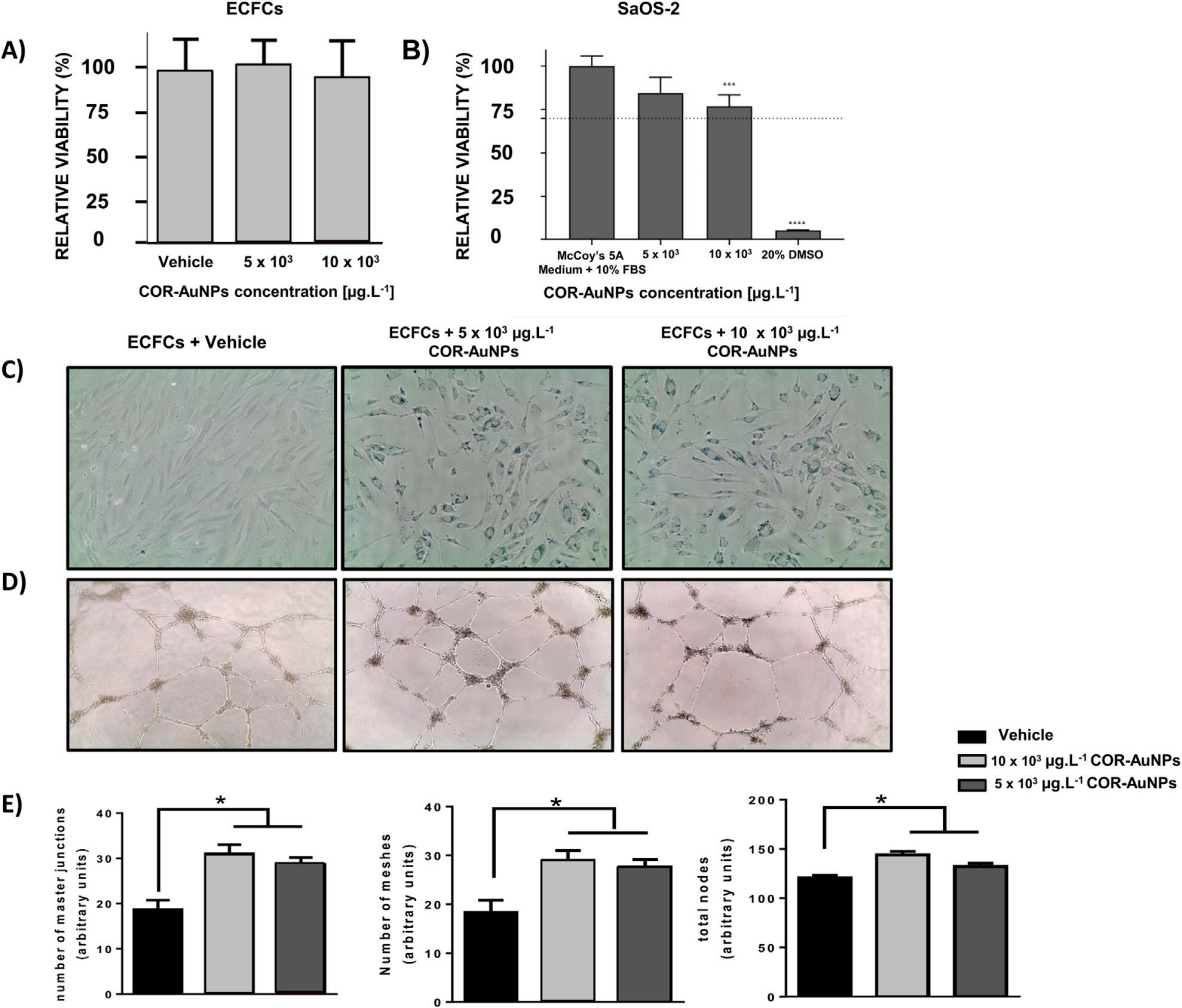
**(A)** Relative viability of ECFCs treated with COR-AuNPs for 24 h, performed by trypan blue assay. For statistical analysis the data were analyzed using GraphPad Prism6 and Origin and expressed as a mean value ± SD. Statistical analysis was performed using One way Anova. **(B)** Relative viability of SaOS-2 cells exposed (24 h) to the COR-AuNPs (5 and 10 × 10^3^ μg.L^−1^) in McCoy’s 5A medium + 10% FBS (vehicle; positive control of cell death (DMSO 20%). The dashed line represents 70% relative viability. Values below this line are considered indicative of cytotoxic effects. Statistically significant differences compared to the controls for each time point are indicated by an asterisks above the bars (***p < 0.0006 and****p < 0.0001). **(C)** Images acquired with optical microscope for ECFCs loaded with COR-AuNPs at different concentrations. The internalized metal loads are identifiable as the dark areas inside the cells (24 h). **(D)** Optical microscopy images of capillary network structures of ECFCs exposed to two different concentrations of COR-AuNPs (upper panel). **(E)** The degree of morphogenesis was quantified using the ImageJ software, by evaluation of the number of master junction, nodes and total networks (meshes) branching out from a branch point/node, as reported in the histograms.

Considering the prospective applications of CO delivery systems in the treatment of vascular diseases (Dulak et al., 2008; Choi et al., 2022), we assessed the cytotoxicity of the nanoparticles in endothelial colony forming cells (ECFCs), a subtype of Endothelial Progenitor cells (Figures 4A,B), as well as in the osteogenic sarcoma cell line SaOS-2.

To further investigate their impact on angiogenesis, a capillary morphogenesis assay was performed using ECFCs. This assay evaluated both nanoparticle uptake (Figure 4C) and their effect on the formation of capillary-like structures (Figure 4D). A clear increase in the number of master junctions, meshes, and master nodes (Figure 4E) was observed. As illustrated in Figures 4A,B, the cells were exposed to vehicle or COR-AuNPs at concentrations of 5 and 10 × 10^3^ μg.L^−1^, and no toxicity was observed after 24 h of incubation. The outcomes can be attributed to the laser synthesis process (Tahir et al., 2024), which does not involve the use of toxic solvent or reagents, and relies exclusively on the physical interaction between light, the target, and the molecules in the liquid environment. These results are consistent with our previous studies on RAW267.4, NCTC and HMVEC cells (Tahir et al., 2024).

In addition to ECFCs, we investigated the effects of COR-AuNPs on SaOS-2 osteosarcoma cells, that did not show cytotoxic potential since the cell solutions offered relative viability greater than 70% in all conditions tested after 24 h of exposure (Figure 4B), when compared to the negative control (untreated cells). Considering that effective bone regeneration is closely linked to neovascularization (Dalle Carbonare et al., 2025), this approach aims to explore the potential applicability of these nanoparticles in regenerative therapies across different tissue types.

### 3.3 Toxicology investigation *in vivo*

#### 3.3.1 Antioxidant biomarkers

The zebrafish model provides additional insights into the *in vivo* effects of COR-AuNPs, particularly concerning oxidative stress, a key parameter in nanotoxicology (Horie and Tabei, 2021). Oxidative stress occurs when the balance between the production of reactive oxygen and nitrogen species (ROS/RNS) and antioxidant defense mechanisms is disrupted (Lushchak and Storey, 2021), which may result in biomolecular harms (Roberts et al., 2010). In this study, we evaluated multiple oxidative stress biomarkers to assess potential disturbances in cellular homeostasis induced by COR-AuNPs exposure.

**FIGURE 5:**
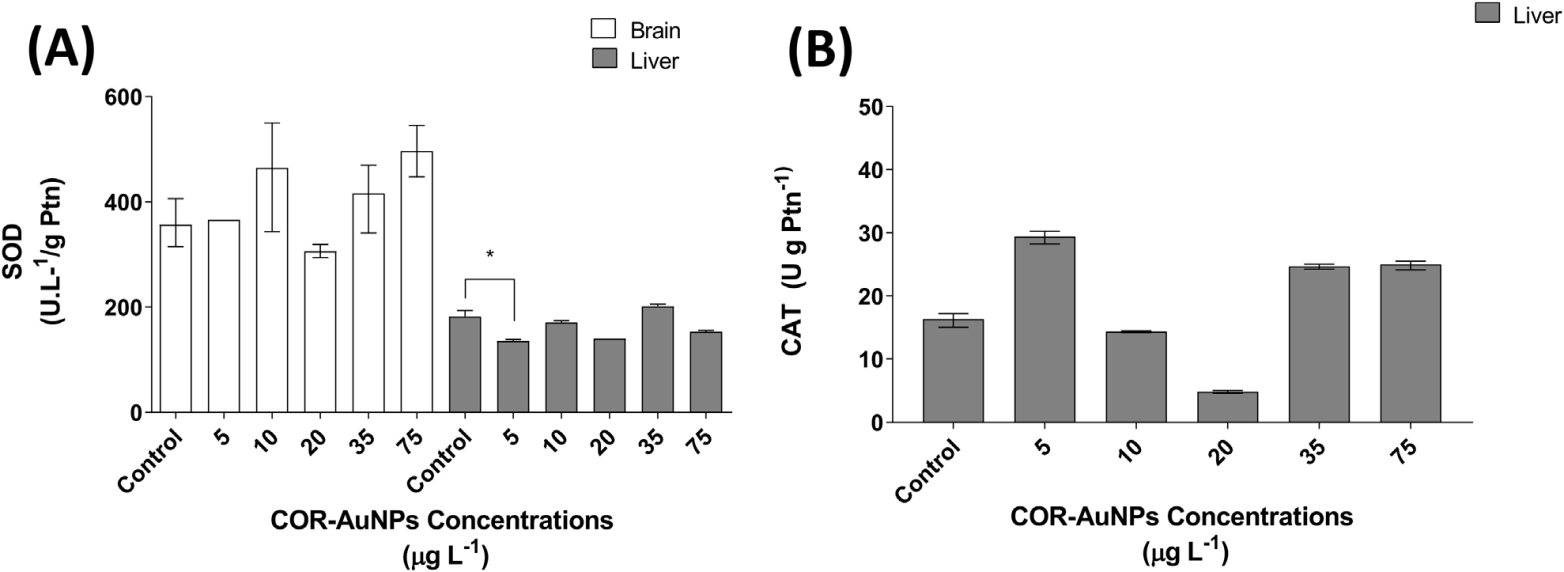
**(A)** Superoxide Dismutase (SOD) enzyme activity in the brain and liver of fish exposed to COR-AuNPs. **(B)** Catalase (CAT) enzyme activity in the liver of fish exposed to COR-AuNPs. Data are presented as median ± standard error of the mean (SEM), based on one independent experiment with triplicates for each experimental group. Statistically significant differences from the control group are indicated by an asterisk (*) above the bars (p ≤ 0.05).

The activity of the enzyme SOD was evaluated in the brain and liver of fish exposed to different concentrations of COR-AuNPs (Figure 5A). No significant changes in SOD activity were observed in the brain at any concentration. In the liver, a substantial decrease in SOD activity was observed at a concentration of 5 µg·L^−1^, while no statistically significant differences were detected at other concentration when compared to the control.. This pattern may suggest reduced bioavailability at higher concentrations, leading to diminished biological effects (Lim et al., 2024). Alternatively, the reduction at 5 µg·L^−1^ could represent a random variation without indication of toxicity.

As ilustrated in Figure 5B below, the activity of the enzyme catalase (CAT) in fish exposed to COR-AuNPs is demonstrated. As reported in the literature, CAT activity is typically reduced in the brains of these animals (Gomes et al., 2019; Araujo et al., 2022), which led us to focus the analysis exclusively on the liver. The stability of these enzymes across most tested COR-AuNPs concentrations suggests that, despite the reduction in SOD activity at lower concentrations, the antioxidant system maintained redox equilibrium (Krishnamurthy et al., 2024). Give SOD catalyzes the dismutation of the highly reactive superoxide radical (O_2_^−^·) into hydrogen peroxide (Zheng et al., 2023), which is subsequently decomposed into water and molecular oxygen by enzymes such as CAT (Anwar et al., 2024), the observed stability in these biomarkers indicates that COR-AuNPs do not significantly disrupt these antioxidant defenses under the tested conditions (Vona et al., 2019). In a previous work, gold nanoparticles synthesized by the seeded growth method (Murphy and Jana, 2002) were assessed for their effects on various biological parameters, including the gene expression of SOD and CAT. The results showed a significant increase in the expression of these enzymes following exposure to the nanoparticles. SOD expression was elevated after exposure to 0.01 nM AuNRs (gold nanorods) (1.97 µg.L^−1^), while CAT expression increased with both 0.01 nM GNRs (1.97 µg.L^−1^) and 0.05 nM PAH-PSS-AuNRs (gold nanorods coated with polyallylamine hydrochloride, PAH) (9.85 µg.L^−1^) (Patibandla et al., 2018). These findings emphasize the influence of nanoparticle shape and coating on toxicity and suggest the low toxicity of the COR-AuNPs synthesized in the present study.

**FIGURE 6:**
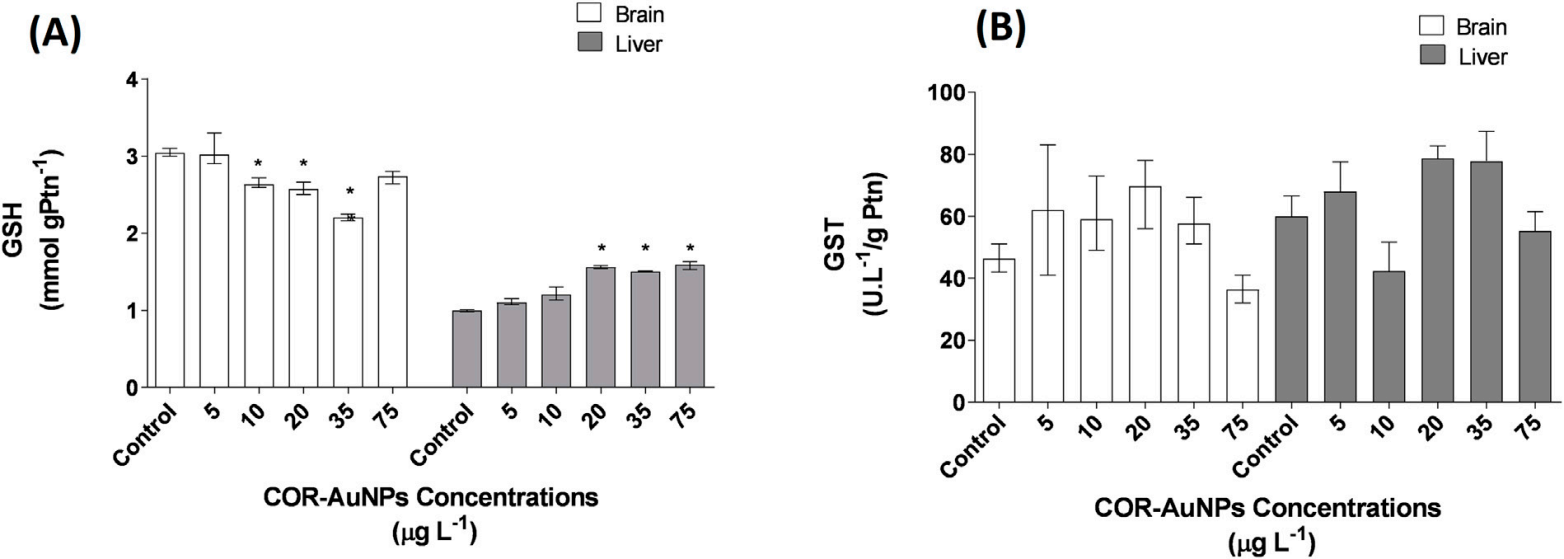
**(A)** Reduced Glutathione (GSH) levels in the brain and liver of fish exposed to COR-AuNPs. **(B)** Glutathione S-Transferase (GST) enzyme activity in the brain and liver of fish exposed to COR-AuNPs. Data are presented as median ± standard error of the mean (SEM), based on one independent experiment with triplicates for each experimental group. Statistically significant differences from the control group are indicated by an asterisk (*) above the bars (p ≤ 0.05).

The levels of reduced glutathione (GSH) were assessed in fish exposed to varying concentrations of COR-AuNPs, exhibiting distinct, tissue-specific patterns. In the brain, GSH levels significantly decreased at concentrations of 10, 20, and 35 µg.L^−1^, while in the liver, a significant increase in GSH levels was observed at concentrations of 20, 35, and 75 µg.L^−1^ (Figure 6A). As the most relevant low molecular weight antioxidant produced within cells (Forman et al., 2009), GSH plays a crucial role in xenobiotic metabolism and in maintaining redox homeostasis, serving as a cofactor for enzymes such as glutathione S-transferase (GST) and glutathione peroxidase (GPX) (Koramutla et al., 2021). The observed modulation of GSH levels suggests that the COR-AuNPs can influence the GSH intracellular levels.

Based on the available scientific evidence, it is plausible that the observed reduction in glutathione levels is related to the direct interaction between gold nanoparticles (AuNPs) and intracellular GSH. The affinity of the thiol (-SH) group in GSH for metals such as gold is well documented, leading to the formation of stable Au–S bonds (Xue et al., 2014; Luo et al., 2016). This interaction may result in GSH depletion, particularly at higher AuNP concentrations, where the organism’s GSH synthesis capacity may become overwhelmed. Additionally, research indicates that small-sized AuNPs can enter cells and preferentially bind to GSH thiol groups, leading to a significant decrease in intracellular GSH levels (Sokolsky-Papkov and Kabanov, 2019; Jawaid et al., 2020). In the context of the present study, the synthesis of COR-AuNPs without thiolated ligands, such as citrate or dihydrolipoic acid (DHLA), and the use of Pluronic F-127 as a stabilizing agent, which does not form covalent bonds with gold, may leave part of the AuNP surface exposed. This exposure facilitates interactions with biomolecules *in vivo*, such as GSH, enhancing the formation of Au–S bonds and contributing to the observed GSH depletion (Xue et al., 2014; Jawaid et al., 2020). Therefore, the hypothesis that GSH is being sequestered by AuNPs, surpassing the organism’s synthesis capacity and leading to reduced GSH levels, is consistent with findings in the literature and may explain the results observed in this study. While the direct interaction of AuNPs with GSH thiol groups appears to be a plausible mechanism for GSH depletion, it is not possible to fully exclude the possibility that the decrease reflects a physiological adjustment due to altered redox demand.

Nevertheless, when considering GSH levels in isolation, it is not possible to determine whether the reduction in this biomarker was triggered by a lack of demand or by a suppression resulting from excessive demand that exceeded the organism’s capacity for GSH synthesis. It has been demonstrated in prior studies that gold nanoparticles produced by the modified Turkevich method (6.3 nm) and capped with dihydrolipoic acid (7.3 nm) posses the capability to modulate GSH levels without inducing ROS generation at concentrations ranging from 1 to 6 µg·L^−1^. While these concentrations differ from those utilized in the present study, they demonstrate that GSH modulation may occur independently of ROS overproduction (Tournebize et al., 2012). Similarly, biologically synthesized AuNPs applied at higher concentrations (9.7–58.2 mg·L^−1^) have been shown to reduce ROS production in zebrafish (Ramachandran et al., 2018). Collectively, these findings lend support to the hypothesis that COR-AuNPs modulate GSH activity as part of an adaptive physiological response, rather than inducing toxicity, even within the concentration range that was evaluated in this study.

On the other hand, increased levels of GSH were observed in the liver at concentrations above 20 µg·L^−1^. This finding may be indicative of an adaptive response aimed at maintaining redox homeostasis. This notion is supported by the established role of GSH in neutralizing reactive oxygen species (ROS) and in the regeneration of other antioxidants through the glutathione redox cycle (Wu et al., 2004). Although the activities of SOD and CAT did not increase to a statistically significant degree, both enzymes demonstrated higher values in comparison to the control group. This trend, though not statistically significant, may suggest a subtle accumulation of reactive oxygen species (ROS) in the liver, which could have triggered a compensatory elevation in GSH synthesis. It is also important to note that increased GSH levels can be beneficial depending on the physiological context, as GSH participates not only in antioxidant defense but also in detoxification processes (Lushchak, 2012) and cellular signaling pathways (Chai and Mieyal, 2023).

Furthermore, the response of Glutathione S-Transferase (GST) activity to these concentrations was investigated. The results demonstrated no statistically significant alterations in GST activity in either the brain or liver, irrespective of the concentration that was examined (Figure 6B). GST plays a pivotal role in the conjugation of glutathione (GSH) with electrophilic species, functioning as a crucial enzyme in the phase II metabolism of xenobiotics (Landi, 2000). However, the results indicated no statistically significant alterations in GST. Conversely, an *in vitro* study revealed that AuNPs did not induce alterations in GST activity within hemolymph and gill cells of *Mytilus galloprovincialis*. It is worth noting that, in the context of co-exposure with pharmaceuticals such as carbamazepine and fluoxetine, AuNPs demonstrated a protective effect, effectively preventing the enzyme induction triggered by these drugs (Barreto et al., 2019). Unlike previous studies reporting GST activity alterations in fish following the exposure of citrate-AuNPs (35 nm) obtained by Turkevich method and functionalized polyvinylpyrrolidon-AuNPs (50 nm) at a concentration of 80 µg.L^−1^ (Luis et al., 2016), our data suggest that the COR-AuNPs synthesized in this study exhibit low biological impact.

**FIGURE 7:**
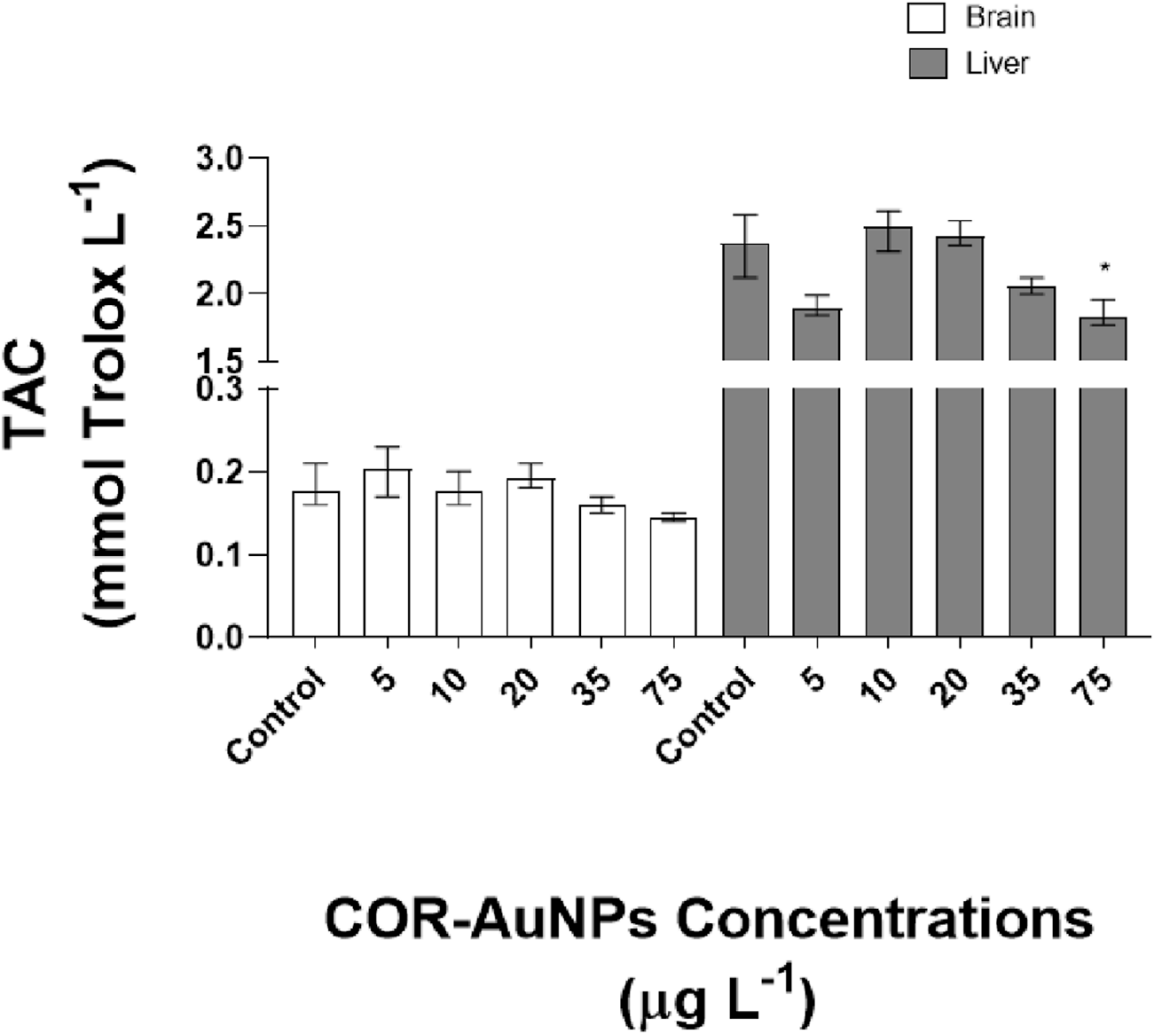
Total Antioxidant Capacity (TAC) in the brain and liver of fish exposed to COR-AuNPs. Data are presented as median ± standard error of the mean (SEM), based on one independent experiment with triplicates for each experimental group. Statistically significant differences from the control group are indicated by an asterisk (*) above the bars (p ≤ 0.05).

Likewise, Total Antioxidant Capacity (TAC), which reflects the synergistic potential of the antioxidant system (Pellegrini et al., 2006) was analyzed in the brain and liver of fish exposed to different concentrations of COR-AuNPs. In the brain, TAC showed no significant variations at any of the evaluated concentrations. In the liver, a significant decrease in TAC was observed at the concentration of 75 μg.L^−1^, while the other concentrations showed values similar to those of the control group (Figure 7). Although this reduction may suggest a potential disturbance in antioxidant capacity at elevated exposure levels, the absence of consistent alterations across other oxidative stress biomarkers limits definitive conclusions. Therefore, when considered in the broader context of this study, the findings do not indicate a pronounced oxidative challenge or impairment of the antioxidant defense system induced by COR-AuNPs.

#### 3.3.2 Oxidative damage indicative biomarkers - CPT and MDA

**FIGURE 8:**
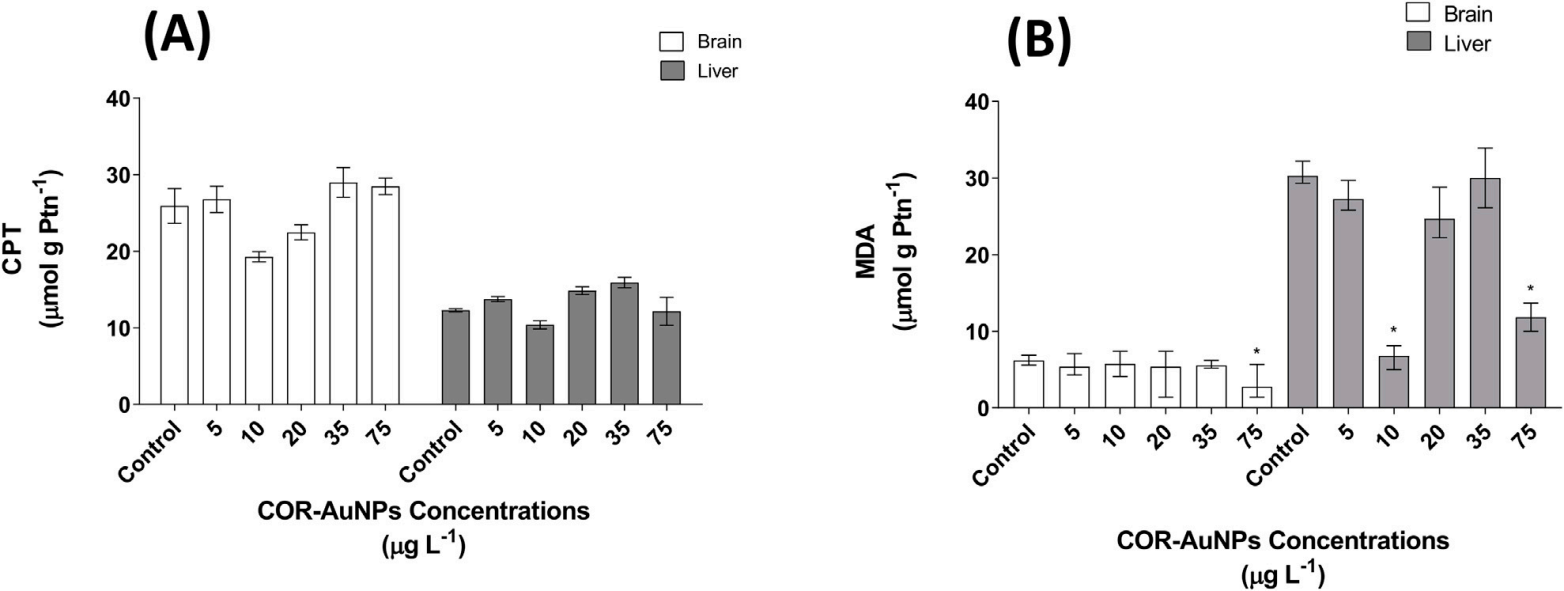
**(A)** Carbonylated Protein (CPT) levels in the brain and liver of fish exposed to COR-AuNPs. **(B)** Malondialdehyde (MDA) levels in the brain and liver of fish exposed to COR-AuNPs. Data are presented as median ± standard error of the mean (SEM), based on one independent experiment with triplicates for each experimental group. Statistically significant differences from the control group are indicated by an asterisk (*) above the bars (p ≤ 0.05).

The levels of Carbonylated Protein (CPT) and malondialdehyde (MDA), both recognized as biomarkers indicative of cellular oxidative damage (Gryszczyńska et al., 2017; Tsikas, 2017), were evaluated to assess their response to different concentrations of COR-AuNPs. The analysis revealed no significant changes in CPT levels in either the brain or liver across the concentrations tested (Figure 8A). Conversely, a significant reduction in MDA levels was observed in the brain at a concentration of 75 μg.L^−1^. In the liver, MDA levels remained stable at most concentrations, with significant decreases occurring at 10 and 75 μg.L^−1^ (Figure 8B), suggesting a potential neuroprotective effect of COR-AuNPs against lipid peroxidation. The present findings are consistent with a prior report indicating that the Turkevich method-synthesized AuNPs can exert a protective effect on the zebrafish nervous system during co-exposure with ethanol, thereby mitigating the solvent-induced increase in SOD, CAT, and acetylcholinesterase activity (Torres et al., 2021). Additionally, treatment with COR-AuNPs has been documented to markedly diminish the levels of SOD, CAT, xanthine oxidase (XO), GSH, and lipid peroxidation products in the brains of fish exposed to bacterial lipopolysaccharides (LPS) (Sadhukhan et al., 2022). Another study that employed zebrafish cells corroborated these findings by demonstrating that the integration of AuNPs synthesized via a chemical route into a graphene matrix with sizes in the range of a few microns decreases its toxicity, oxidative stress, and aggregation, thereby enhancing the stability of these nanomaterials in biological systems after exposure to 1, 50, and 100 µg mL^−1^. Notably, the highest concentration exhibited a significant reduction compared to AuNPs alone and the graphene oxide-AuNPs hybrid material (Ibrahim et al., 2025).

#### 3.3.3 Genotoxicity

**FIGURE 9:**
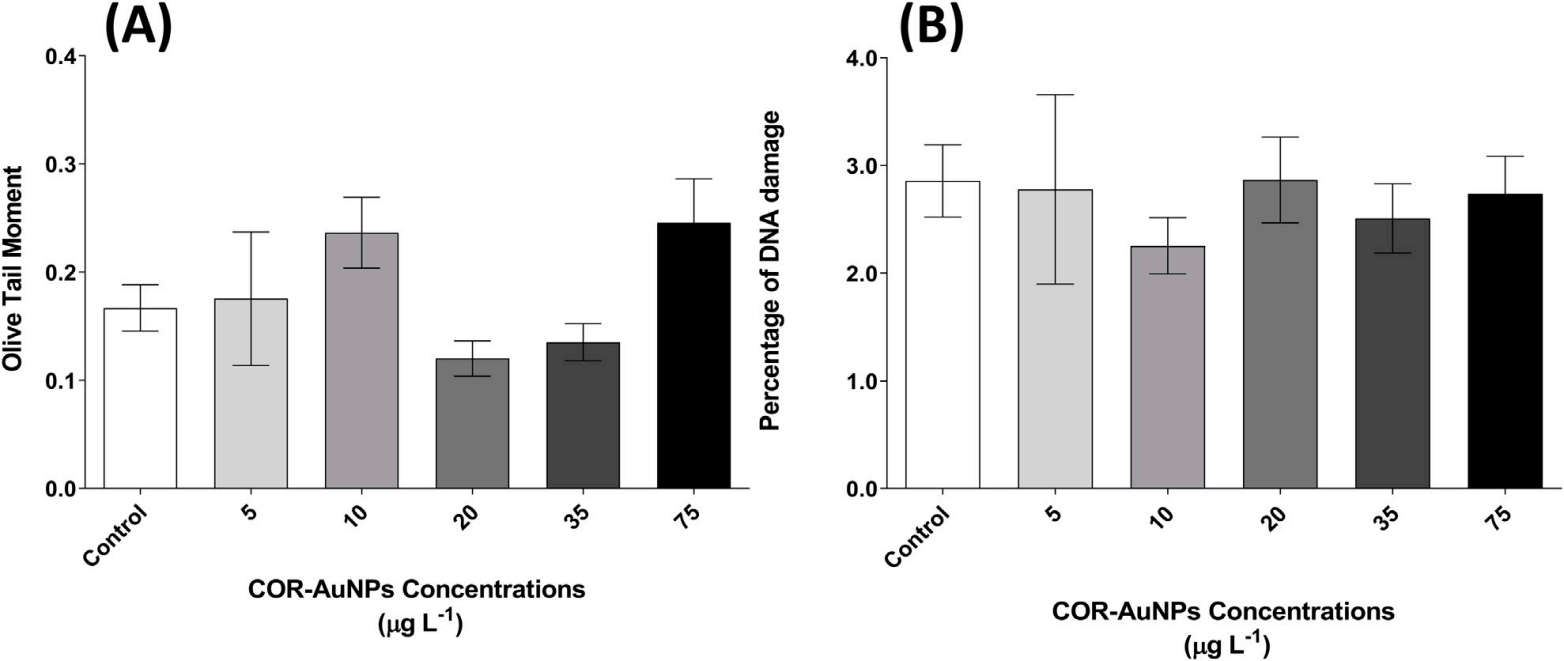
Genotoxicity evaluation in zebrafish blood cells exposed to COR-AuNPs using the comet assay. **(A)** Olive Tail Moment and **(B)** percentage of DNA in the comet tail. Data are expressed as median ± standard error of the mean (SEM) from one independent experiment conducted in triplicate for each experimental group. No statistically significant differences were observed compared to the control group at any tested concentration. Statistically significant differences, when present, are indicated by an asterisk (*) (p ≤ 0.05).

According to the comet assay results, no evidence of DNA damage was observed at any of the tested concentrations of COR-AuNPs, considering regularly previous negative control of organisms not exposed to any contaminants, and positive exposed to methylmethanesulfonate (MMS) at a concentration of 0.4 mM and 0.8 mM (Supplementary Figure S1 on Supplementary Material). This absence of effect was demonstrated by the stable DNA fragmentation levels in the blood cells of the exposed animals, with no increase in Olive Tail Moment (Figure 9A), which reflects the extent of the damage, (Kumaravel et al., 2009), or in tail damage percentage (Figure 9B). These findings are consistent with a previous study (Ramachandran et al., 2018), which also reported no genotoxicity in adult zebrafish from biologically synthesized AuNPs with size and concentration in the range of 30 nm, and 9.7–58.2 mg.L^−1^ respectively, using the micronucleus assay. It is notable that the capacity of these nanoparticles to release CO may also contribute to the lack of genetic toxicity, as CO has been demonstrated to exert a protective effect on DNA in cells (Juszczak et al., 2020).

The COR-AuNPs synthesized in this study demonstrated a well-defined spherical morphology and maintained high colloidal stability throughout the experimental period. Our findings reinforce the biocompatibility of these nanoparticles, as no cytotoxic effects were observed in the ECFCs and SaOS-2 cells at none of the concentrations used. Moreover, COR-AuNPs did not induce genotoxicity at any of the tested concentrations. The stability of DNA fragmentation levels in the blood cells of exposed animals confirms the absence of detectable genetic damage under the experimental conditions. The remarkable findings regarding biocompatibility and low toxicity in the biological assays of laser-synthesized COR-AuNPs in cells and in the zebrafish accessed in this work can be related to the synthesis procedure (Tahir et al., 2024), the absence of toxic solvents, with use of solely light-matter interaction, as well as the surrounding atmosphere, inducing the CO_2_RR (Tahir et al., 2024), resulting in the production of COR-AuNPs and organometallic gold based clusters rich in carboxylic acids. Since CO can be used as a protective agent (Ryter et al., 2018), it could be a contributing factor to the stability of oxidative stress biomarkers, absence of significant protein damage, potential neuroprotective effects and lack of genotoxicity, highlighting the safety profile of these nanoparticles. Despite their extensive potential for application in biomedicine, including the delivery of pharmaceuticals (Abu-Dief et al., 2021), antineoplastic therapy (Kumar et al., 2012), antimicrobial action (Li et al., 2014), nanosensors (Hou et al., 2023), and disease diagnostics (Mieszawska et al., 2013), COR-AuNPs hold great promise for future clinical and technological advancements (Milan et al., 2022).

### 3.4 Literature comparison

The application of metallic nanostructures in biological models (cells and animals) has generated a substantial body of literature, often with conflicting findings due to variations in synthesis methods and experimental models. The use of different concentration ranges in *in vitro* and *in vivo* assays reflects model-specific sensitivities and endpoints. Furthermore, it is essential to consider the physicochemical properties of the nanoparticles, which are inherently determined by the synthesis method. Table 1 provides a direct comparison between the synthesis methods used in prior studies and the current approach.

**TABLE 1:** Comparison of different synthesis methods for AuNPs, highlighting key characteristics, limitations, and implications for biological studies. The table critically evaluates chemical, ionic liquid-based, biogenic, and pulsed laser ablation in liquid (PLA) approaches in terms of synthesis yield, and reproducibility.

Synthesis method	Key characteristics	Limitations	Implications for biological studies	References
Chemical (e.g., Brust–Schiffrin)	High synthesis yield; relatively good control over size and morphology	Use of toxic organic solvents and reducing agents (e.g., toluene); extensive purification required	Residual contaminants may interfere with bioassays; reduced biocompatibility	Brust et al. (1994)
Ionic Liquid-based	Greener approach; potential for colloidal stability without conventional surfactants	High cost; potential toxicity of residual salts; limited scalability	Requires toxicity assessment of residual components; limited reproducibility	Kim et al. (2004)
Biogenic (plant/microbe)	Environmentally friendly; does not require toxic reagents	High polydispersity; limited control over morphology and surface chemistry	Batch-to-batch variability; standardization challenges; risk of biological interference	Shankar et al. (2004), Noruzi et al. (2011)
PLA	Possibility to control surface chemistry of the AuNPs by CO_2_RR; high chemical purity of the core of the NPs; environmentally friendly; does not require toxic reagents	Requires specialized equipment (laser source); lower yield compared to chemical methods	Lowers risk of interference agents; excellent biocompatibility; suitable for biomedical and toxicological applications	Del Rosso et al. (2018), Tahir et al. (2024)

PLA was chosen for this study due to its unique synthesis process, which does not require the use of reagents or surfactants. This distinguishes it from other chemical methods, such as the Brust–Schiffrin protocol (Brust et al., 1994), or ionic liquid-based syntheses (Kim et al., 2004), which do require the use of these substances. These methods still require complex purification to remove residual chemicals, in contrast to the relatively uncomplicated process involved in PLA synthesis.

In comparison with biologically mediated synthesis routes, PLA offers superior reproducibility and better control over key synthesis parameters, including particle size distribution, morphology, and chemical composition. This is particularly especially pronounced in the context of biogenic approaches, which frequently yield nanoparticle formulations that are characterized high polydispersity, a broad spectrum of morphological variants, and inconsistent surface chemistry. For instance, Noruzi et al. (2011) and Shankar et al. (2004) reported the formation of heterogeneous mixtures of gold nanostructures when using biological extracts as reducing and capping agents.

Conversely, laser ablation in liquids facilitates the direct synthesis of nanoparticles in a pristine environment, circumventing the introduction of extraneous contaminants and ensuring greater batch consistency, a prerequisite for biomedical and toxicological investigations.

Furthermore, the physical and chemical characteristics of the nanostructure have been demonstrated to exert a substantial influence on its biological response when exposed to cells or organisms. It has been observed that surface chemistry plays a pivotal role in determining the biological effects of AuNPs. Studies on cellular responses has shown that commonly used stabilizers, such as citrate, exhibit negligible toxicity in most cases (Connor et al., 2005; Mironava et al., 2010; Tsai et al., 2013), whereas charged surfactants, particularly those with a positive charge, significantly reduce biocompatibility (Schaeublin et al., 2011). For instance, AuNPs functionalized with CTAB (cetyltrimethylammonium bromide) exhibited higher levels of cellular toxicity in comparison to gold nanorods (AuNRs) in both the Human Dermal Fibroblast and U87 cell lines (Bhamidipati and Fabris, 2017). Furthermore, when CTAB-stabilized AuNRs were exposed to HeLa cells, they exhibited severe toxicity, which was mitigated by replacing the surfactant (Mallick et al., 2013). These findings underscore the dominant role of surface ligands and charge in modulating the biocompatibility of AuNPs.

A comparable trend is evident in zebrafish models, where charged surfactants have been associated with augmented toxicity (Truong et al., 2013). The morphology of AuNPs has been demonstrated to play a significant role in their toxicological profile, particularly at *in vivo* models. In zebrafish, exposure to non-spheroidal nanoparticles resulted in a more pronounced toxic response compared to exposure to spherical particles (Patibandla et al., 2018; Souza et al., 2021). While analogous patterns are discerned in cell models, *in vivo* systems manifest a heightened sensitivity to structural disparities. A number of studies have suggested that AuNPs may exert neuroprotective effects, while others have reported embryotoxicity, including morphological abnormalities and impaired development (Patibandla et al., 2018; Verma et al., 2018; Torres et al., 2021). These findings suggest that nanoparticle shape can significantly influence biological interactions, especially during early developmental stages.

The impact of particle size on the toxicity of AuNPs is a complex matter, as there is a divergence of opinions on the subject. Some evidence has suggested that larger nanoparticles induce greater cytotoxic effects (Mironava et al., 2010), while others have suggested that smaller nanoparticles exhibit higher toxicity due to their greater surface area and reactivity (Broda et al., 2016). These discrepancies underscore the intricacy of size-dependent effects and the impact of confounding variables, such as surface chemistry, agglomeration state and synthesis method. Despite the presence of conflicting findings regarding morphology and size, a clear and consistent trend across studies is the dose-dependent increase in cytotoxic effects. It has been observed that elevated concentrations of AuNPs frequently correlate with augmented toxicity, both cellular and systemic (Connor et al., 2005; Mironava et al., 2010; Schaeublin et al., 2011; Tsai et al., 2013; Broda et al., 2016; Bhamidipati and Fabris, 2017). In zebrafish, for instance, exposure to AuNP resulted in both dose-dependent toxicity and tissue-specific bioaccumulation in the brain, muscles, gills, and gastrointestinal tract, evaluated over a 60-day period (Geffroy et al., 2012). This accumulation may be associated with long-term toxic effects, including modulation in the expression of genes related to DNA repair and detoxification pathways (Geffroy et al., 2012; Dedeh et al., 2015). Consequently, further studies with the COR-AuNPs synthesized in the present work are warranted to evaluate longer exposure periods and potential cumulative effects.

Another relevant aspect concerns the selection of sublethal endpoins. Torres et al. (2021); Sadhukhan et al. (2022) reported that AuNPs can modulate endogenous antioxidant defenses and influence the activity of key enzymes in zebrafish. Importantly, (Torres et al., 2021), observed that these parameters returned to baseline after exposure, indicating a transient and reversible sublethal biological response (Torres et al., 2021). In line with these findings, our study also focused on sublethal endpoints, evaluating sensitive oxidative stress biomarkers and early indicators of cellular damage, including lipid peroxidation (MDA), protein carbonylation (PTC), and DNA damage (comet assay), and found no significant alterations following acute exposure to COR-AuNPs.

This study offers a significant advancement in the field by assessing the toxicity of gold nanoparticles produced by pulsed laser ablation, enriched with oxocarbons, in zebrafish. Notably, the synthesis approach employed in this research is devoid of chemical contaminants, a notable feature that enhances the study’s rigor and reliability. In the literature, Gong et al. (2016) reported that cobalt-based CORMs did not induce significant toxicity in zebrafish embryos at concentrations below 1.0 µM (equivalent to about 60.0 µg·L^−1^ of Co) Gong et al. (2016). Comparatively, in the present study, COR-AuNPs were tested at concentrations ranging from 5 to 75 µg·L^−1^, without causing mortality or significant alterations in oxidative stress and genotoxicity biomarkers. These results reinforce that CO-releasing nanomaterials, when properly stabilized and free of toxic ligands, may exhibit a safe profile at concentration ranges similar to those previously established for cobalt-based CORMs.

Therefore, the present study demonstrates that COR-AuNPs produced by pulsed laser ablation exhibit high biocompatibility in zebrafish, with no significant acute toxicity or sublethal effects observed at the concentrations that were tested. These results are consistent with previous findings for cobalt-based CORMs and highlight the critical influence of synthesis method, surface chemistry, and particle size on the biological responses to metallic nanoparticles. Furthermore, subsequent studies should encompass conventional gold nanoparticles devoid of CO-release properties to distinguish the effects attributable to CO from those of the nanoparticle core.

## 4 Conclusion

This study provides consistent evidence of angiogenic responses of the COR-AuNPs to treated ECFCs. The safety profile of the laser-synthesized COR-AuNPs was thoroughly demonstrated, showing low cytotoxicity to human cells, limited effects on oxidative stress biomarkers, and absence of genotoxicity in zebrafish, a well-established model for human-related toxicological and biomedical research. These findings underscore the potential of COR-AuNPs for biomedical applications, particularly in light their clean synthesis route, free from surfactants and undesired residual ligands, which distinguishes them from conventionally produced AuNPs. It is imperative to note, however, that the biological behavior of the COR-AuNPs is intimately associated with their physicochemical characteristics and the experimental parameters of the synthesis method, which have the potential to vary, for instance, in terms of their CO content. Consequently, while the results obtained herein provide valuable insights into the biocompatibility of this specific nanomaterial, further studies are necessary to explore its long-term effects, potential for bioaccumulation, and responses under varying physiological conditions, thereby supporting the safe and effective use of COR-AuNPs in future biomedical applications.

## Data Availability

The raw data supporting the conclusions of this article will be made available by the authors, without undue reservation.
