# Supplementary material for: Toxicity evaluation of laser-synthesized pro-angiogenic carbon monoxide-rich gold nanoparticles in vitro and in vivo
Source: Front Bioeng Biotechnol. 2025 Nov 26;13:1594693. doi: 10.3389/fbioe.2025.1594693 (PMC12689510; doi:10.3389/fbioe.2025.1594693)
Supplement: Supplementary file 1 [file Supplementaryfile1.docx]

Supplementary Material

Table S1. Minimum Information for Reporting on the Comet Assay (MIRCA) for genotoxicity assessment of COR-AuNPs

| **Parameter** | **Description** | **Value/Description** |
| --- | --- | --- |
| Organism | Zebrafish (Danio rerio) | Adult fish, 6 per group |
| Sample type | Blood cells | Collected by puncture of the caudal vein |
| Number of cells scored | Minimum 50 cells per slide, 5 slides per group | ≥ 200 cells per group |
| Positive control compound | Methyl methanesulfonate (MMS) | 0.4 mM and 0.8 mM |
| Negative control compound | Aquarium water only | - |
| DNA damage quantification metrics | Tail intensity, Olive Tail Moment, Tail length, Score (0 to 4 scale per Noroozi et al., 1998) | Used as described in Results and Discussion. |
| Electrophoresis conditions | Alkaline buffer (NaOH 10 mol/L, EDTA 0.2 mmol/L), 25 V, 300 mA, 20 min DNA unwinding + 20 min electrophoresis | Temperature 0–4 °C |
| Slide preparation | Low melting point agarose embedded cells on NMP-coated slides | Solidification at 4 ± 2 °C for 10 min |
| Staining | Silver nitrate | Standard staining procedure |
| Data analysis software | Motic Image Plus 2.0® | Quantitative image analysis |

Figure S1. DNA damage in zebrafish blood cells exposed to MMS (0.4 and 0.8 mM): comet tail DNA percentage and tail moment.


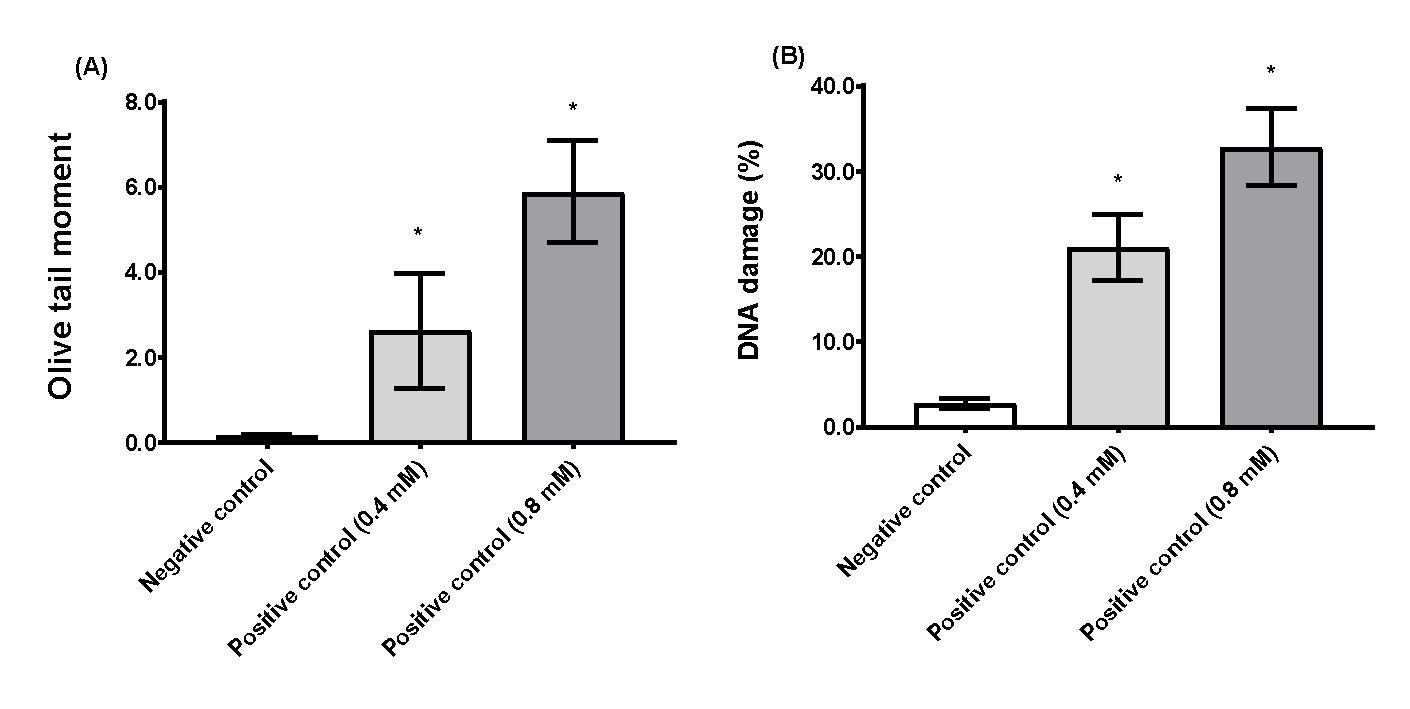


Figure S1: Percentage of DNA in the comet tails of blood cells from zebrafish exposed to positive control group composed of organisms exposed to methylmethanesulfonate (MMS) at a concentration of 0.4 mM and positive control group composed of organisms exposed to methylmethanesulfonate (MMS) at a concentration of 0.8 mM.


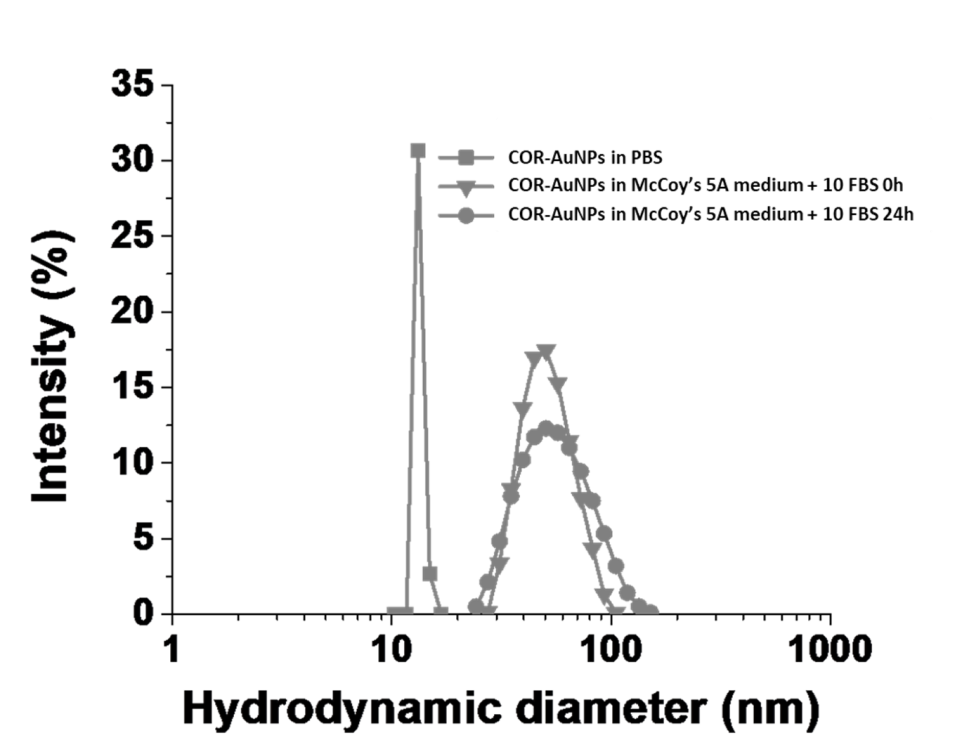


Figure S2: Hydrodynamic diameter of the COR-AuNPs in PBS at 0h (square points) and in cell culture medium at 0h (triangular points) and after 24h (circular points).
